# A Survey on Satellite Communication System Security

**DOI:** 10.3390/s24092897

**Published:** 2024-05-01

**Authors:** Minjae Kang, Sungbin Park, Yeonjoon Lee

**Affiliations:** 1 Major in Bio-Artificial Intelligence, Department of Computer Science and Engineering, Hanyang University, Ansan 15588, Republic of Korea; minjae0110@hanyang.ac.kr (M.K.); pbt98@hanyang.ac.kr (S.P.); 2 College of Computing, Hanyang University, Ansan 15588, Republic of Korea

**Keywords:** satellite communication system, security attack methodologies, security countermeasure, space policy and strategy

## Abstract

In recent years, satellite communication systems (SCSs) have rapidly developed in terms of their role and capabilities, promoted by advancements in space launch technologies. However, this rapid development has also led to the emergence of significant security vulnerabilities, demonstrated through real-world targeted attacks such as AcidRain and AcidPour that demand immediate attention from the security community. In response, various countermeasures, encompassing both technological and policy-based approaches, have been proposed to mitigate these threats. However, the multitude and diversity of these proposals make their comparison complex, requiring a systemized view of the landscape. In this paper, we systematically categorize and analyze both attacks and defenses within the framework of confidentiality, integrity, and availability, focusing on specific threats that pose substantial risks to SCSs. Furthermore, we evaluate existing countermeasures against potential threats in SCS environments and offer insights into the security policies of different nations, recognizing the strategic importance of satellite communications as a national asset. Finally, we present prospective security challenges and solutions for future SCSs, including full quantum communication, AI-integrated SCSs, and standardized protocols for the next generation of terrestrial–space communication.

## 1. Introduction

The Internet of Everything (IoE), which aims to connect all devices and systems in every possible location, has become the next dominant objective of communication. However, traditional land-based communication systems (e.g., cellular communication) are unable to cover the vast distances required for such connections. For instance, agriculture management with smart devices in the US requires vast coverage because of its land area, which existing telecommunications cannot cover. In response, satellite communication systems (SCSs) have become a popular alternative due to their wide coverage.

Although SCSs have an advantage with respect to coverage, in the early days, the use of SCSs was restricted to military and scientific applications, such as surveillance and weather forecasting [1], due to the high costs associated with the technical advancement of satellite launches and communication between satellites. However, with the advancement of both technologies, as exemplified by SpaceX [2,3] and Radio Frequency (RF)-based communication, SCSs have evolved significantly in recent years.

**Figure 1:**
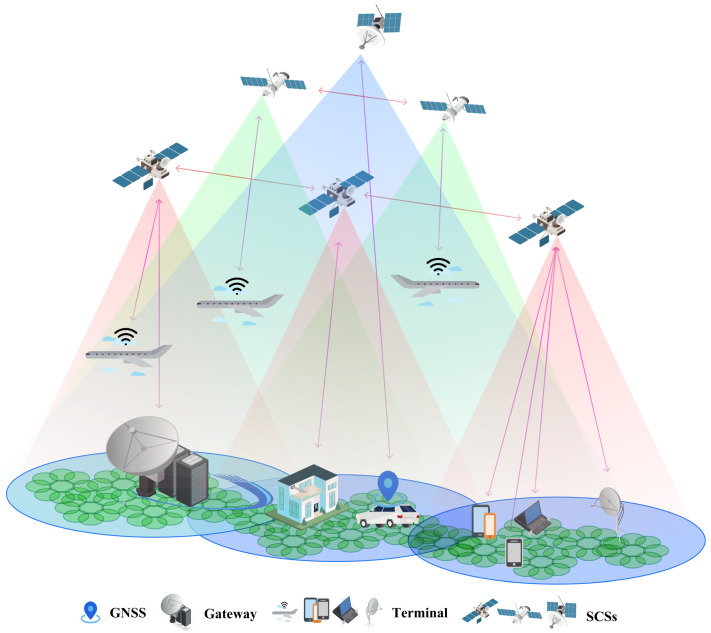
Satellite communication system scheme.

Rapid development has resulted in various consequences. One is user experience enhancement. For instance, companies like OneWeb and Amazon Kuiper provide low-latency and wide-coverage communication by integrating mega-constellation low Earth orbit (LEO) SCSs with ground communications (through gateways or direct communication with a terminal). This technology enables communication in places where ground communication alone is inadequate, such as aircraft, ships, and rural areas, and provides users with a similar communication experience as in cities (Figure 1). Another is enhancing the performance of existing technology. For example, in the past, the global positioning system (GPS) created by the United States was the only option available. Nowadays, consumers can access more precise time and position data through various ground navigation satellite systems (GNSSs) powered by SCSs, such as GLONASS, Galileo, and Beidou, developed by countries or alliances (Figure 1).

However, the extensive development and deployment of SCSs have not come without challenges. Cyber attacks on SCSs have increased due to the exploitation of vulnerabilities that stem from their environmental and technical limitations. For example, military drone traffic information during the Iraq War, communicated via an SCS, was eavesdropped due to the lack of encryption caused by the limited communication bandwidth (compared to terrestrial communication) of the SCS [4]. Also, in 2011, Iran conducted a GPS spoofing attack on a U.S.-built RQ-170 Sentinel that misled a UAV into landing in Iran by feeding it false GPS information, making it perceive Iran as its home base in Afghanistan. This is because the significant distance between Earth and satellites results in communication delays, fostering latency and increasing the susceptibility of SCSs to spoofing threats.

In addition to attacks on dedicated SCSs, cyber attacks on the latest SCS for general communication are also prevalent. For example, during the early stages of the Russia–Ukraine war, the deployment of the AcidRain [5] malware targeted Ukraine’s KA-SAT satellite broadband service, causing widespread disruption by wiping data for thousands of SCS modems across Ukraine and Europe. This attack not only rendered the modems inoperative but its impact also extended to the remote monitoring or control of thousands of wind turbines. Following this, a new variant of the malware, named AcidPour, was discovered, demonstrating an evolution in the cyber threat landscape targeting satellite communication systems. The progression from AcidRain to AcidPour highlights cyber threats to satellite communications, underscoring the requirement for robust defense mechanisms to protect these critical infrastructure components from such destructive attacks.

Not only has KA-SAT been an attack target, but so has Starlink, the most commercialized SCS for general communication, during the ongoing conflict between Ukraine and Russia, which exposed many threats. For example, SpaceX, the company that operates Starlink, has reported instances of jamming attacks on Starlink terminals in Ukraine. In response, they have updated the system’s software to counter such threats [6]. Additionally, recent research has also shown that Starlink terminals can be compromised by using a custom modchip to execute arbitrary code via voltage fault injection, which bypasses signature verification [7].

To address these emerging threats and ensure the reliability and security of global communications, numerous studies have been proposed. These efforts aim to deal with physical layer security [8,9,10], improve cryptographic security measures [11,12,13], and integrate terrestrial–space network frameworks [14,15,16,17,18]. However, there is still a lack of systematic categorization and a limited knowledge about SCSs’ security issues in existing research.

## 2. Existing Surveys and Motivation

In this section, we summarize and compare existing survey papers, presenting their works and limitations. We then introduce the motivation for our research and, in the final part, outline the overall structure of our research.

### 2.1. Existing Surveys

As the importance of understanding SCSs grows, various kinds of research concentrating on specific aspects have been published. We categorize the existing works into three categories.

First, some studies deal with satellite security issues in the physical layer (PHY). Mucchi et al. [8] compile security techniques in the PHY based on Physical Layer Security (PLS). They present solutions to counterattacks like eavesdropping. However, a limitation of this research is that it only briefly addresses attacks and solutions specific to SCSs. Li et al. [9] address terrestrial- and space-integrated network architectures and present their PLS-based solutions. This study summarizes that properly designed interference or jamming can be used to enhance the secrecy performance of legitimate users in satellite–terrestrial integrated networks (STINs), and also focuses on the PLS that has been applied to strengthen secure transmission in a cognitive STIN (CSTN), where the secondary terrestrial system shares downlink spectral resources with the primary satellite system. This approach has shown that interference from terrestrial networks can improve the secrecy performance of satellite users. However, this study did not present solutions other than security in the PHY and did not provide detailed methods for attacks that can occur in SCSs. Lu et al. [10] focus on the PHY security of UAVs in 6G systems based on reinforcement learning (RL); they also delve into RL-based frameworks for safeguarding security and privacy, demonstrating the application of RL algorithms in optimizing security policies for 6G. This research includes strategies for security resource allocation and tuning authentication parameters to counteract various threats such as jamming, eavesdropping, spoofing, inference attacks, and selfish attacks.

Second, some studies explore alternative approaches to address security problems related to specific types of SCSs. Wang et al. [11] also categorize threats such as false data injection and denial of service (DoS) and distributed denial of service (DDoS) attacks into five categories and propose methods to build a reliable and trustworthy network using blockchain technology. However, they did not provide solutions for security in the PHY, such as defenses against spoofing or jamming attacks. Wang et al. [12] focus on methods for converging terrestrial and satellite networks based on software-defined networks (SDNs)/network function virtualization (NFV). Since satellite communications are conducted through wireless channels, which are open and thus vulnerable to issues like eavesdropping, this paper presents methods to address this challenge. However, it does not mention other threats beyond eavesdropping, nor does it provide solutions for them. Hosseinidehaj et al. [13] focus on a new security framework in SCSs, QKD, which is significant for its potential to fundamentally prevent attacks like eavesdropping. It comprehensively covers research from the traditional optical fiber-based QKD to the recent advancements in wireless-based QKD. However, these studies may address some solutions for specific attacks and not provide enough solutions for other areas.

Third, some papers present new space–ground integrated networks and address both the security threats and solutions associated with them. Guo et al. [14] address security issues across various layers including terrestrial, maritime, submarine, and space. They discuss potential threats and solutions in these layers, as well as natural disasters that can occur within this communication framework. Vaezi et al. [15] focus on the wireless communications used in Internet of Things (IoT), categorizing potential attacks in IoT wireless communications into physical, software, and network areas. They provide an analysis of deep learning (DL)-based methods for the IoT. However, the study scarcely covers attacks related to SCSs and their corresponding solutions. Yue et al. [16] address the overall security and reliability issues in LEO satellite systems, offering solutions for these challenges. They also cover physical threats, which were previously under-addressed. However, this study only covers general attacks and solutions related to SCS networks and is not divided in detail according to clear classification criteria. Tedeschi et al. [17] address the security threats, solutions, and challenges associated with the deployment and operation of SCSs, particularly at the link layer. This paper categorizes schemes into two principal domains: PLS and cryptographic schemes. Manulis et al. [18] present a comprehensive analysis of the cybersecurity threats facing the space industry, especially in the context of the emerging New Space era. It assesses past security threats and incidents to understand adversarial threats to satellites, with a focus on the ground and RF communications as primary targets. However, they did not clearly present countermeasures against attacks. In Table 1, we compare and summarize the major attack and defense and main research points of each study.

### 2.2. Motivations

Although some existing works have tried to address various issues of SCSs, more work is needed to provide a comprehensive understanding of the SCS landscape.

Insufficient Review Papers on Attacks and Defences for general SCSs: Numerous studies have tackled the potential risks and remedies associated with special-purpose SCSs. However, these studies do not provide a comprehensive overview of the entire SCS landscape. Furthermore, the existing research that discusses specific attacks or defenses lacks clear classification standards and detailed descriptions of the threats and solutions in SCSs.

Lack of Discussions on Various Aspects of SCSs: Previous studies on SCSs have mainly focused on their technological aspects. However, SCSs are also a national asset, and it is essential to discuss their non-technological aspects, including policy and physical aspects.

In terms of policy, conducting policy analyses regarding cyber attacks in SCSs is crucial as they can significantly impact future research. Unfortunately, existing works have not addressed these issues, so more research is needed. On the other hand, when it comes to physical aspects, anti-satellite (ASAT) attacks are the most common attacks due to various countries’ recent policy movements and geopolitical situations. However, no existing work has addressed the ASAT landscape, so we need to make an effort to understand it.

### 2.3. Contributions

The contributions of this survey are below.

- We provide a comprehensive understanding of SCSs, including the background and attack and defense mechanisms based on intuitive criteria (i.e., confidentiality, integrity, and availability).
- From our understanding, we are the first to provide the non-technical aspects of space security, such as countries’ security policies and the physical ASAT landscape.
- We present future research directions, including potential security issues arising from future SCSs, such as full quantum networks.

### 2.4. Paper Organization

**Figure 2:**
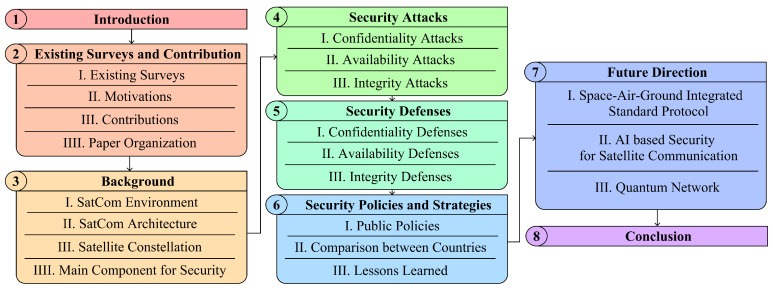
Paper organization.

The organization of this paper is illustrated in Figure 2. Section 3 addresses the background of SCSs and their environment. Section 4 introduces security attacks in SCSs. Section 5 describes the security defense method for attacks on SCSs. Section 6 presents security policies and strategies in space and compares each country. In Section 7, future directions for research are provided. Lastly, we summarize our research in the conclusion and provide the abbreviations that are used in this paper.

## 3. Background

This section covers the background knowledge related to SCSs. First, we will address the overall architecture of SCSs, including the basic hardware payload that satellites carry. We then deal with the unique environmental aspects of SCSs, as well as the characteristics of frequency and bandwidth specific to SCSs. Furthermore, we discuss the main protocols used in satellites and the features of space-to-ground integrated networks. The above content includes key concepts that will be used later when discussing threats and solutions.

### 3.1. Satellite Communication Environment

SCS environments are fundamentally different from terrestrial-based communication networks. This is primarily because satellites operate continuously in the unique conditions of space. While this special environment brings many advantages to SCSs, it also introduces various disadvantages. Therefore, in this section, we will examine the environmental aspects that need to be considered in SCSs [19].

**Vacuum:** The space environment is essentially a vacuum. The boundary between the Earth’s atmosphere and outer space, commonly referred to as the Kármán line, is defined as being approximately 70–90 km above the Earth’s surface. Most SCSs orbit at a minimum altitude of 200 km in LEO or higher, meaning they are essentially in a vacuum. This vacuum state has a variety of impacts on satellites [20].

**Gravity:** The zero gravity state is a condition with minimal resistance. This significantly affects satellites, whether they are subjected to external forces or generate internal torque. For example, Earth’s gravity is a factor to consider. The gravitational field of the Earth primarily determines the motion of the satellite’s center of mass. This gravitational field is not linear and uniform across all regions, leading to orbital perturbations. Additionally, the gravitational fields of the Sun and Moon can cause disturbances. Moreover, the strength of the Earth’s gravitational field varies with altitude, and as a result of this gravitational gradient, the force does not pass through the satellite’s center of mass. This means that this effect can generate torque within the satellite itself.

**Solar Radiation Pressure:** Solar radiation pressure, although a very weak force, can significantly affect a satellite’s orientation in a resistance-free environment due to its continuous influence. The solar radiation pressure can be considered as a force that arrives perpendicularly from the sun to the satellite. Typically, the resultant force of solar radiation does not align with the satellite’s center of mass. Consequently, this misalignment generates torque that can disturb the satellite’s orientation.

**Torques of Internal Origin:** Satellites consist of various components, and the movement of parts like antennas, solar panels, and fuel can generate torque on the satellite. For instance, as the propellant is consumed, the center of mass of the satellite may shift, and adjusting the direction of the satellite’s antenna can induce torque on the satellite’s body. Therefore, to compensate for these effects, forces acting on the satellite’s center of mass need to be periodically applied.

### 3.2. Satellite Communication System Architecture

**Figure 3:**
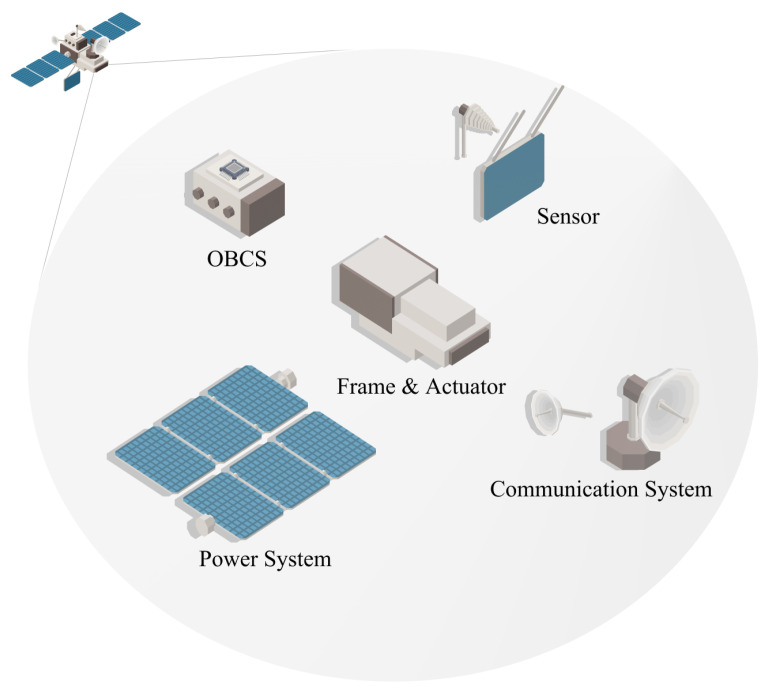
Satellite architecture.

Generally, a satellite’s hardware is specific for its purpose. In Figure 3, the five types of payload generally used in satellite systems are shown [21,22].

**Onboard Computer System:** The onboard computer system (OCS) is a central component that controls the satellite and is often referred to as the satellite’s processor [23]. The satellite’s OCS plays a crucial role in coordinating various sensors and hardware components. Firstly, the processor controls the sensors contained within the satellite’s payload, and it performs computations to determine the satellite’s orientation and position by adjusting actuators. Additionally, the OCS handles the encryption and decryption of data packets during communication.

**Sensors:** Sensors play a fundamental role in the operation of satellites, performing key functions such as measuring the velocity, acceleration, orientation, and tracking position. Examples of these sensors include antennas, solar sensors, star trackers, Earth sensors, inertial units, RF sensors, laser detectors, angular rate sensors, magnetometers, and temperature sensors. In addition to these, there are special-purpose sensors that, unlike the ones listed above, do not influence the basic operation of the satellite but are included in the satellite payload for specific objectives. These special-purpose sensors often include cameras, SAR (Synthetic Aperture Radar), and Light Detection and Ranging (LiDAR) systems [24].

**Actuators:** Actuators play a primary role in controlling the orientation of the satellite and the direction of its sensors. Actuators are crucial in space environments due to the virtually resistance-free nature of space, meaning satellites continue to move under the influence of inertia. In other words, even weak forces such as solar wind can alter a satellite’s orientation or altitude, necessitating actuators for correction. For instance, in communication satellites, precise antenna positioning is crucial [21], and actuators are used to counteract the inertia moment. Typically, actuators produce torque to change angular velocity, but there are also actuators that use propellants. Key components of these actuators include angular momentum devices, thrusters, magnetic coils, and solar sails.

**Power System:** The power system is a component that supplies energy to the satellite. Since satellites are limited in mass and volume, it is essential to precisely design the power system to fit the payload. The power system not only supplies electricity to sensors, the OCS, and actuators, but also needs to be precisely controlled and managed to avoid issues like power shortages and system downtime [25]. In the past, onboard power supply devices like nuclear fission devices were used, but in recent times, especially in power-intensive satellite systems like SCSs, external power supply devices such as solar panels are almost exclusively used. Solar panels are typically used in conjunction with batteries, which serve as auxiliary power supplies, ensuring the availability of power even when solar radiation is not available.

**Communication System:** The communication payload is the component used for communication in satellites. It is utilized for various spatial communications, including ground-to-ground, satellite-to-satellite, and satellite-to-ground communication. The primary components of a communication payload typically include repeaters and antennas [26].

### 3.3. Satellite Constellation Characteristics

In SCSs, the constellation is one of the most crucial elements. The orbit of a satellite greatly influences the area it can cover, which is also linked to the number of satellites. For example, as the satellite’s orbit lowers, the propagation delay and error rate decrease, but the area a single satellite can cover on the ground is significantly reduced. A solution to this is deploying satellites that can fully cover the globe. However, this increases construction and maintenance costs considerably. Thus, a satellite’s orbit has a significant impact on various aspects of the SCS network. In this section, we will explore the different orbits used in SCSs. Also, Table 2 summarizes the satellite constellation features [27].

**Geostationary Earth Orbit:** This orbit matches the Earth’s rotational speed with the satellite’s angular velocity. This means the satellite always faces the same region of the Earth’s surface, which is an advantage utilized by various satellites. Geostationary orbits operate at a latitude of 0 degrees, directly above the equator, and are typically positioned at an altitude of approximately 36,000 km. Additionally, the typical communication latency is between 125 ms and 250 ms, resulting in a delay of nearly 0.5 s for communication between ground stations and satellites.

The advantages of geostationary orbit (GEO) include, firstly, the satellite appears stationary from a specific point on Earth, which is advantageous for providing communication and broadcasting services. Secondly, a single GEO satellite can cover about one-third of the Earth’s surface, allowing services to be provided over a wide area. Thirdly, because it is relatively stationary, the communication delay is consistent. Lastly, once Earth terminals or ground stations adjust their antennas, they can maintain a continuous connection with the satellite.

However, there are disadvantages as well. Firstly, GEO satellites must be placed in high orbits, which increases launch costs and the technical complexity. Secondly, the longer travel time for communication signals to and from the satellite can result in a relatively high communication latency and potential data loss. Thirdly, GEO satellites are bigger and more expensive to deploy; the network operator can gradually add to their coverage as their business grows. Lastly, due to their fixed position relative to the Earth’s surface and the Earth’s tilt, GEO satellites struggle to cover polar regions [28].

**Medium Earth Orbit:** This orbit is located between 9000 and 11,000 km and is situated within the Van Allen radiation belt. MEO is at a lower altitude compared to geostationary orbit. Primarily, this orbit is used for navigation satellite systems like GPSs [27].

The advantages of medium Earth orbit (MEO) include, firstly, shorter communication delay times compared to GEO satellites, as MEO satellites operate in a lower orbit. This also allows MEO satellites to cover a wider area than LEO satellites, while requiring fewer satellites, thereby reducing system costs and complexity.

However, there are disadvantages. Firstly, the communication delay is higher compared to LEO satellites, which can pose challenges for real-time communications. Secondly, satellites are visible for only 2 to 8 h from Earth, necessitating satellite tracking, due to their rotation. Lastly, signals become weaker when reaching the Earth compared to LEO, requiring more transmit power.

**Low Earth Orbit:** This orbit refers to a range approximately 180 to 2000 km above the Earth’s surface. In this orbit, there are various types of satellites, and due to the characteristics of LEO, it is primarily used in high-speed SCSs.

The advantages of LEO are as follows. Firstly, its proximity to the Earth results in a lower communication latency, which is advantageous for real-time communications. Additionally, this closeness to Earth aids in enhancing data transmission speeds. Secondly, LEO satellites are in a lower orbit compared to GEO satellites, which reduces the cost per launch. Lastly, satellites in LEO are capable of collecting higher-resolution images and data [29].

However, there are disadvantages to LEO orbit. First, due to the low altitude of LEO satellites, they can only cover a small portion of the Earth at a time, necessitating a large number of satellites for global coverage. This requirement for a vast number of satellites increases the complexity and cost of the network. Second, LEO satellites are more susceptible to atmospheric drag, which can result in a shorter lifespan. Finally, the LEO orbit is crowded with space debris, posing a heightened risk of collisions.

### 3.4. Main Components for Security

The three pillars of security are Confidentiality, Integrity, and Availability, known as CIA, the most fundamental elements in safeguarding data within cybersecurity. Failing to protect or inadequately secure any aspects exposes them to attacks or threats that compromise the CIA triad. Therefore, this study categorizes attacks and defenses according to the CIA framework, with detailed discussions in subsequent sections [30].

**Confidentiality:** Confidentiality generally refers to ensuring that information is accessible and comprehensible only to authorized individuals. It plays a crucial role in preventing unwanted disclosure of information, closely linking it to privacy protection. The most common form of attack related to confidentiality is eavesdropping, which is particularly prevalent in SCSs due to the wireless nature of their transmission. SCSs that are not encrypted due to technical limitations pose significant threats to confidentiality. Encryption is the best method to prevent such breaches, but it may not be sufficient. Additional measures are necessary and will be elaborated on later.

**Integrity:** Integrity signifies that only authorized individuals have the access and ability to modify information. Integrity is compromised when unauthorized persons alter or access the information. In SCSs, spoofing is a primary method of compromising integrity. Spoofing involves transmitting forged signals to deceive the receiver, thereby breaching integrity. Protective measures include PLS and encryption, which ensure the integrity of communications and enhance their reliability.

**Availability:** Availability denotes the assured, timely access and usability of information or systems. This implies that information or systems must be properly provided and functional whenever needed to be considered secure regarding availability. SCSs can face availability issues due to different factors such as jamming and DoS/DDoS attacks. A compromise in availability can render SCSs unusable, posing a significant threat, especially in emergencies. Ensuring availability requires traffic control through authentication systems like blockchain or filtering unauthorized signals using PLS.

## 4. Attacks in Satellite Communication Systems

**Figure 4:**
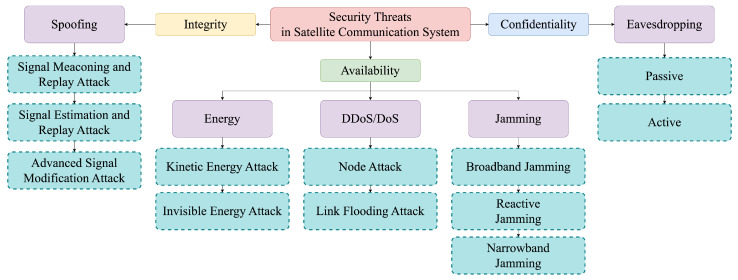
Attack schemes in satellite communication systems.

As SCSs continue to evolve, they play a crucial role in both civilian and military purposes. Consequently, various forms of attacks are being directed at SCSs. In this section, we will explore attacks on SCSs, with the overall structure depicted in Figure 4.

### 4.1. Confidentiality Threats

Eavesdropping

**Figure 5:**
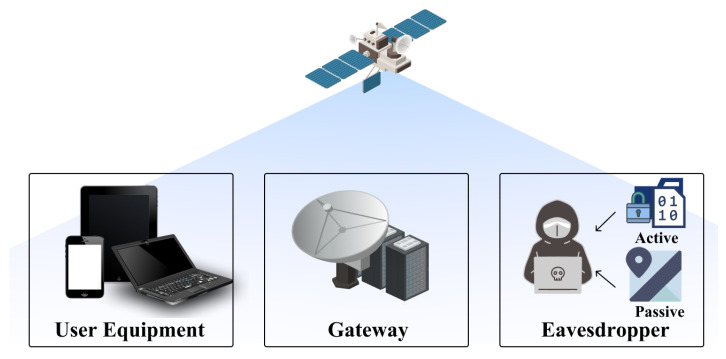
Eavesdropping attack scheme.

Eavesdropping entails the secretive act of intercepting and listening to private conversations, electronic communications, or transmissions between individuals without their knowledge or consent, often using sophisticated devices or techniques to breach confidentiality. This type of attack is traditionally associated with overhearing conversations without being detected, but in the modern context, it extends to various forms of electronic and digital surveillance [31]. An eavesdropping attack in SCSs is a type of security breach where an unauthorized party intercepts and listens to the communication transmitted via a satellite network. This has become possible due to the characteristics of satellites, such as wireless communication, wide coverage, and unencrypted communications resulting from a low bandwidth. Also, this kind of attack can be particularly concerning due to the broad coverage and diverse use of SCSs, ranging from personal communication to critical military and government transmissions. The attack involves equipment to intercept the satellite signal ranging from a simple setup for unencrypted signals to more complex systems capable of breaking encryption. In this section, we approach eavesdropping attacks by dividing them into passive eavesdropping and active eavesdropping. Additionally, the overall process of the two attacks is depicted in Figure 5 and a comparison of the two attacks is presented in Table 3.

**Passive Eavesdropping:** Passive eavesdropping is listening to or recording communication without altering or interacting with the transmission. The eavesdropper is essentially a silent observer. This type of attack can be difficult for the victim to detect, but it may also be challenging for the attacker to obtain meaningful information.

The authors of [32] introduced RECORD, a novel passive attack method targeting LEO satellite users which compromises their location privacy by exploiting the downlink from wandering communication satellites. This method, implemented on a custom satellite reception platform, uses real-world data from the Iridium SCS to demonstrate that observing just 2.3 h of traffic can significantly narrow down a user’s position to less than an 11 km radius, a drastic reduction from the initial 4700 km satellite beam diameter. Also, [33] presents a detailed experimental security analysis of satellite broadband signals using the Digital Video Broadcasting for Satellite (DVB-S) protocol, highlighting vulnerabilities that allow for the identification of individual satellite customers and their activities, as well as posing threats to critical infrastructure. This research uncovers various network topologies prone to interception, revealing a substantial amount of sensitive data, including Secure Sockets Layer (SSL) and Transport Layer Security (TLS) certificates, unencrypted Hypertext Transfer Protocol (HTTP) requests, emails, and Voice Over Internet Protocol (VoIP) conversations. This data exposure raises severe risks to critical infrastructure, especially in power generation facilities where unencrypted communications were detected.

**Active Eavesdropping:** Active eavesdropping involves a certain level of intervention in communication. It is an attack where the eavesdropper decrypts encrypted signals or intervenes in the communication process to directly intercept the data. While this form of attack increases the risk of detection, it also requires the collection of more effective information.

Pavur et al. [34] introduced a breakthrough in decrypting communications within the Thuraya satellite network, which uses the GEO-Mobile Radio(GMR)-1 standard. By enhancing a ciphertext-only attack and leveraging open-source software, the authors successfully decrypted their own communications, showcasing vulnerabilities in the GMR-1 and GMR-2 SCS standards. Utilizing common, moderately priced equipment, they captured and decrypted a live call session from the Thuraya network in under an hour using standard PC hardware. This was achieved by exploiting weaknesses in the A5-GMR-1 cipher and solving a system of linear equations to recover the session key. Also, ref. [35] presents an approach for eavesdropping on optical communications between LEO satellites and High-Altitude Platform Stations (HAPSs), focusing on both downlink and uplink transmissions. It investigates the secrecy performance of these communications, particularly under scenarios where an eavesdropping spacecraft is positioned close to the satellite within its optical beam’s convergence area. This study, validated through Monte Carlo simulations, reveals that downlink communications are generally more secure, with increased beam leakage to the eavesdropper significantly impairing the secrecy performance.

### 4.2. Availability Threats

- Jamming

**Figure 6:**
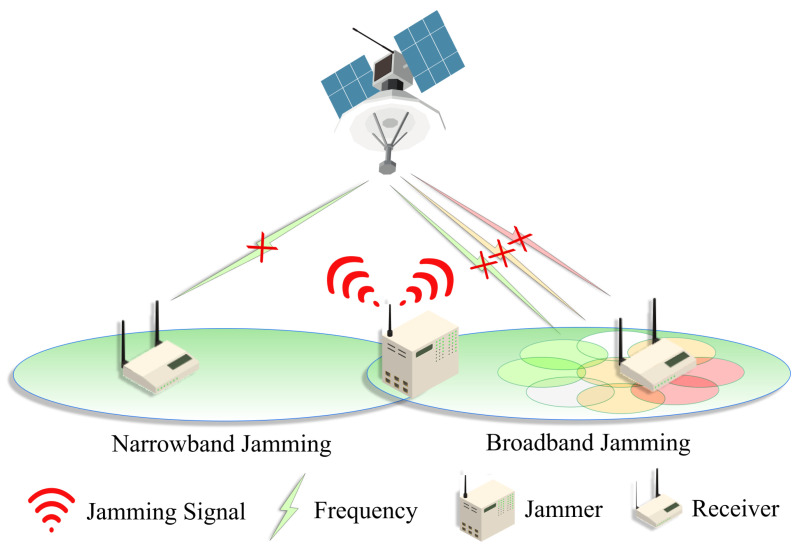
Illustration of narrow and broadband jamming attacks.

A jamming attack in an SCS is a deliberate interference with satellite signals. It involves transmitting signals at the same frequencies used by the satellite, thereby overwhelming the legitimate signals with noise or other forms of interference. This can degrade, obstruct, or prevent successful communication via the satellite. This is because an SCS relies on wireless RF signals, which are inherently weak and can be easily overwhelmed. The goal of jamming can range from simple disruption of communication to more complex strategies aimed at deceiving or manipulating the satellite system’s operations. The techniques used in jamming can vary, including broadband and narrowband jamming, each with its specific method of disrupting SCSs. Also, various forms of attacks are illustrated in Figure 6.

**Broadband Jamming:** This type of jamming covers a wide range of frequencies simultaneously. It is designed to disrupt communication by flooding multiple frequency bands with noise or false signals. Broadband jammers are less precise but can be effective against a wide array of communication systems at once.

The authors of [36] investigate the impact of jamming on the GPS and the GLONASS system, particularly in maritime navigation. They offer a detailed analysis of how varying the jamming intensities affects these satellite navigation systems, noting a decrease in the carrier-to-noise ratio and precision in pseudorange measurements, leading to a compromised positioning accuracy. This study finds the acquisition phase of receivers more vulnerable to interference than the tracking phase. Notably, under jamming conditions, GLONASS signals perform better than those of the GPS. This research included both static and dynamic jamming tests to explore the effects on different GPS receivers. Similarly, ref. [37] investigates the vulnerabilities of the GPS in maritime navigation, particularly focusing on the effects of GPS jamming. It was found that GPS jamming significantly impacts maritime safety by affecting on-shore vessel traffic services, aids-to-navigation (AtoNs), ship navigation, situational awareness, and emergency communications. Lichtman et al. [38] explore reactive jamming in SCS scenarios. Reactive jamming is characterized by a jammer’s ability to sense a portion of the spectrum and transmit a jamming signal upon detecting a signal it wants to disrupt. This technique can counter the processing gain associated with the Frequency-Hopping Spread Spectrum (FHSS).

**Narrowband Jamming:** Narrowband jamming focuses on disrupting specific frequencies or a narrow range of frequencies. This method is more precise and is used when the frequency of the target communication is known. It is effective at blocking specific channels without affecting others.

Ref. [39] focuses on jamming attacks against GPS receivers using Direct Sequence Spread-Spectrum (DSSS) signals. It highlights the GPS’s vulnerability to intentional and unintentional interference due to its weak signal strength, which can be easily overpowered. A key aspect of this paper is its proposal of a cascade filter approach for countering multiple frequency modulation (FM) jammers, especially effective at high jammer-to-signal ratios. This approach prioritizes the signal-to-noise ratio as the performance measure and treats FM interference as instantly narrowband.

b. DoS and DDoS

DoS and DDoS attacks are malicious efforts aimed at disrupting the normal functioning of a targeted server, service, or network. A DoS attack typically involves a single device or computer generating malicious traffic or actions to achieve its goals, such as seizing control of equipment or rendering it unusable. In contrast, DDoS attacks use multiple devices or computers to generate malicious traffic or actions. This often involves the use of botnets to infect multiple devices, allowing a collective of individual attackers to target a single objective.

**Figure 7:**
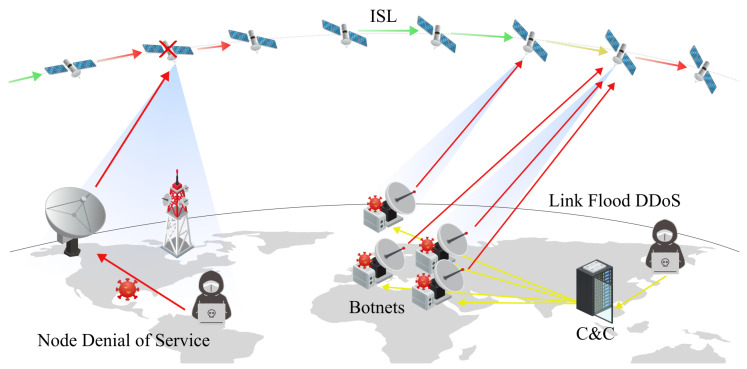
DoS and DDoS attacks in a satellite communication system.

In SCSs, DoS and DDoS attacks usually involve attackers sending inappropriate traffic to specific nodes (satellite equipment) to make them inoperative or to hijack them. These attacks can also consume the entire bandwidth of SCS links, degrading the quality of service (QoS) of the satellite network. This section will approach the topic by differentiating between DoS and DDoS attacks. In Figure 7, the differences between the most representative forms of DoS and DDoS attacks are shown.

**Denial of Service Attack:** A DoS attack typically achieves its objective, such as seizing or rendering a device unusable, using a single device or computer initiating malicious traffic or actions.

In [40], the authors demonstrate the vulnerability of satellite systems to attacks using software-defined radio (SDR). This research is crucial as it shows that individuals with basic equipment like SDRs can disrupt SCSs. This study focused on the Reaktor Hello World satellite, which, despite encrypted command and control channels, is susceptible to a replay attack due to unencrypted telemetry data and the absence of authentication between the satellite and the ground station. Using simple tools like a Linux laptop and a HackRF device, the researchers successfully executed the attack, underlining the need for enhanced security in SCSs. Onen et al. [41] highlight the susceptibility of satellite shared access networks, particularly DVB-S, to DoS attacks. These attacks are characterized by exploiting the network control center through numerous fraudulent requests from a satellite terminal. Their paper outlines three primary forms of these attacks: (1) Consuming scarce, limited, or non-renewable resources like network bandwidth, memory, or CPU processing power, which impairs the service availability for legitimate users. (2) Destroying or altering configuration information, leading to ineffective network or computer use for legitimate users due to corrupted or tampered data. (3) Physically damaging or altering network components, such as cables, routers, or servers, resulting in service degradations or complete shutdown. This study underscores the critical need for robust security measures to protect SCS networks from these types of vulnerabilities. In addition, Ref. [42] introduces a method for executing ransomware attacks on space vehicles, particularly those utilizing NASA’s core flight system (cFS). The proposed attack method strategically targets the software bus API of the cFS, a component critical to the command and data handling functionalities and mission-specific applications. This approach is designed to compromise the spacecraft’s operations without permanently disabling it, maintaining a balance between compelling the victim to pay the ransom and ensuring the possibility of restoring the spacecraft’s functionality after the ransom is paid.

**Distributed Denial of Service Attack:** A DDoS attack is a malicious attempt to disrupt the normal traffic of a targeted server, service, or network by overwhelming the target or its surrounding infrastructure with a flood of internet traffic. DDoS attacks are conducted by utilizing multiple compromised computer systems as sources of attack traffic.

Normally, there are three common types of DDoS attacks: (1) TOS (type of service) floods, where attackers manipulate the TOS field in IP headers, which is used for explicit congestion notification (ECN) and differentiated service (DiffServ) flags. Attackers spoof the ECN flag, reducing the throughput of individual connections and making a server appear non-responsive or attackers utilize DiffServ class flags in the TOS field to prioritize attack traffic over legitimate traffic, intensifying the DDoS attack’s impact. (2) IoT botnet attacks, which involve infecting numerous IoT devices to form botnets for large-scale, hard-to-detect attacks, exemplified by the Mirai botnet. (3) Ping floods, where attackers flood networks with spoofed internet control message protocol (ICMP) echo requests, causing significant slowdowns or complete service blackouts. Paper [43] highlights the increasing risks of DDoS attacks on satellite service providers, exacerbated by the growing number of internet-connected infrastructure devices. They studied the above three types of attacks. Similarly, ref. [44] primarily contributes to the field by presenting a DDoS technique that faces unique challenges due to its inherent characteristics like a high bit error rate, high link delays, power control, and large round trip delays, and presents the same types of attacks as the aforementioned paper, including TOS floods, SYN floods, and ICMP floods.

The paper by Giacomo Giuliari [45] focuses on exploring a new class of distributed denial-of-service attacks, a type of link flooding attack, on LEO satellite networks, specifically targeting their routing mechanisms. The ICARUS attack, targeting LEO satellite networks, involves a series of steps to disrupt communication by congesting specific network links. Initially, the attacker discovers the network topology and calculates routes between compromised nodes in a botnet, focusing on paths that intersect targeted links. These paths are then filtered to ensure they traverse these links in the desired direction. The attacker calculates the optimal traffic volume to cause congestion without self-interference, distributing this traffic across multiple uplinks to minimize detection. In cases of networks with randomized multipath routing, the attack adapts to a probabilistic approach, increasing the chances of congesting the target links despite the uncertainty in path selection. This method showcases how even a limited number of compromised hosts can significantly impact LEO satellite network communications.

c. Energy Attack

The concept of a kinetic attack involves rendering satellites inoperable through physical, mechanical, or invisible energy like a laser. This is perhaps the most definitive and clear-cut method to compromise the availability of satellites. Energy attacks have been a subject of design and research since the onset of the space arms race. Such attacks are referred to as ASAT weapons and their forms have become increasingly varied over time.

During the Cold War, the intensifying space race led the United States and the Soviet Union to recognize the potential military significance of satellites. Consequently, both nations pondered countermeasures against satellites and conducted various experiments. Early ASAT efforts were not significantly different from missile defense systems. This similarity stemmed from the fundamental resemblance between ICBMs and space launch vehicles. Initially, the U.S. equipped missile interceptors with nuclear tips due to the lack of advanced guidance systems.

As the 1960s progressed, the Soviet Union introduced the co-orbital ASAT weapon. This system involved launching a weapon into the same orbit as the target satellite and maneuvering it close enough to destroy the target, usually with conventional explosives. In response to the U.S. ASAT technology, the Soviet Union proposed a new type of attack in the 1970s, the Terra-3 [46], a high-energy laser weapon. This weapon was designed to incapacitate enemy satellites using laser beams.

The 1980s saw the U.S. develop a new-generation ASAT weapon, the ASM-135A ASAT, consisting of a missile launched from an F-15 fighter to intercept and destroy satellites using a kinetic kill approach. Although not deployed in actual combat, this weapon successfully destroyed the Solwind P78-1 satellite during tests. In response to the AGM-135 ASAT, the Soviet Union modified the Mig-31 Foxhound. However, clear experimental evidence of its effectiveness was not available. Later in the 1980s and into the 1990s, the U.S. began developing new ground-based energy weapons. A notable project was the MIRACL [47] (mid-infrared advanced chemical laser).

Entering the 2000s, new nations joining the space race introduced additional threats. China, rapidly advancing in space technology, developed various interception systems, notably the SC-19 missile. This system was a mobile ground-based missile that launched a homing vehicle to destroy satellites via direct impact. China conducted a satellite destruction test using the SC-19 on the FY-1C satellite [48] and successfully developed a submarine-launched ASAT weapon, the 2 missile. The U.S. also started developing various weapon systems in response to these new threats. American ASAT experiments and interception systems, such as the RIM-161 Standard Missile 3 (SM-3), were developed. The U.S. successfully conducted an actual satellite interception test, Operation Burnt Frost, with the SM-3 [49]. After the Soviet Union collapsed and Russia was established, Russia developed the new laser weapon Peresvet. It exhibited significant achievements in the recent Ukraine conflict, although the U.S. dismissed these claims as propaganda. Russia also developed the Burevestnik [50], a new missile potentially ready for deployment, evolving from the Foxhound missile.

**Figure 8:**
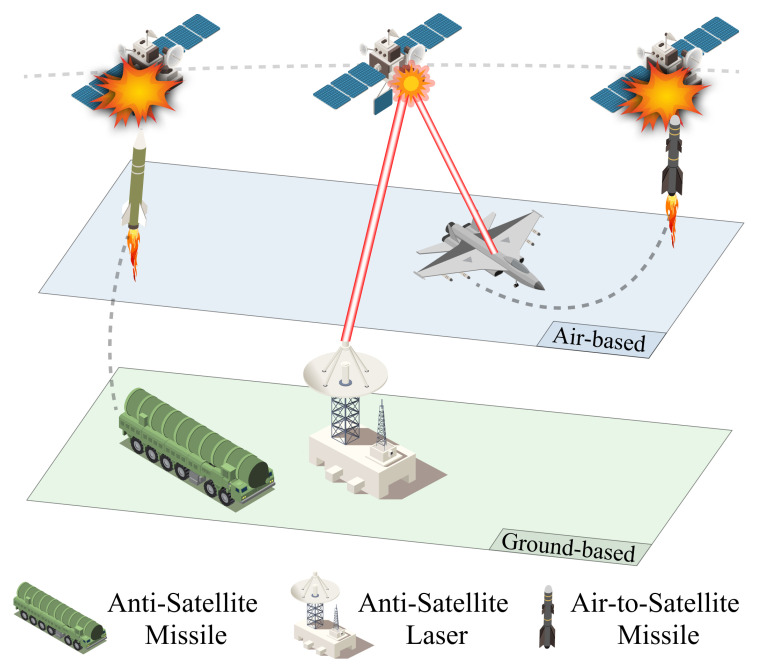
Illustration of ASAT technology.

In summary, various forms of satellite interception systems have been developed, which can be summarized as follows. Also, the overall ASAT types can be seen in Figure 8.

**Kinetic Energy Attack:** This method involves destroying target satellites by colliding high-speed moving objects with them. There are different types of kinetic ASAT weapons, including direct-rising ASAT missiles and common-orbit satellite interceptors. These weapons use rocket propulsion to accelerate a warhead to high speeds for a direct collision or to generate dense metal fragments to destroy the target in space. Prominent weapon systems include the United States’ SM-3, Russia’s Burevestnik, and China’s SC-19.

**Invisible Energy Attack:** These attacks use high-energy beams like lasers and microwaves to neutralize or destroy a target. Directed energy weapons have the advantage of a fast attack speed and can potentially reach the speed of light. Paper [51] discusses high-energy laser beam ASAT weapons, high-energy particle beam ASAT weapons, and high-frequency microwave radio-frequency ASAT weapons, elaborating on their technological aspects and capabilities. Some notable examples include the United States’ MIRACL and Russia’s Peresvet.

### 4.3. Integrity Threats

#### Spoofing

Satellites, due to their significant distance from the ground and reliance on wireless signals, often transmit weak signals that are susceptible to interference. This vulnerability allows for various forms of attacks, among which spoofing can compromise the integrity of the signal. Spoofing involves deceiving a target or multiple target receivers by delaying, forging, or sending incorrect signals [52]. Conceptually, while spoofing and jamming may seem similar, spoofing is a more sophisticated and technical attack. Both attacks target the availability of satellites, but spoofing requires intricate signal processing, making it a more complex attack to execute.

Spoofing fundamentally requires replicating the RF carrier signal transmitting the pseudo-random noise and spreading code, and the data from GNSS. Generally, the signals transmitted from a GNSS are of the form:

(1) $$y\left(t\right)=Re\left\{\sum_{i=1}^{N}A_{i}D_{i}\left[t-\tau_{i}\left(t\right)\right]C_{i}\left[t-\tau_{i}\left(t\right)\right]e^{j[\omega_{c}t-\phi_{i}\left(t\right)]}\right\}$$

where $A_{i}$ is the carrier amplitude of signal, $D_{i}\left(t\right)$ is the signal’s data bit stream, $C_{i}\left(t\right)$ is the spreading code, $\tau_{i}\left(t\right)$ is the signal’s code phase, $\omega_{c}$ is the nominal carrier frequency, and $\phi_{i}\left(t\right)$ is the beat carrier.

At this time, the spoofing signal is in a form similar to the original signal:

(2) $$y_{s}\left(t\right)=Re\left\{\sum_{i=1}^{N_{s}}A_{si}{\hat{D}}_{i}\left[t-\tau_{si}\left(t\right)\right]C_{i}\left[t-\tau_{si}\left(t\right)\right]e^{j[\omega_{c}t-\phi_{si}\left(t\right)]}\right\}$$

and each spoofed signal has the same N and $C_{i}\left(t\right)$ and usually sends estimated data of the ${\hat{D}}_{i}\left(t\right)$ [53].

**Figure 9:**
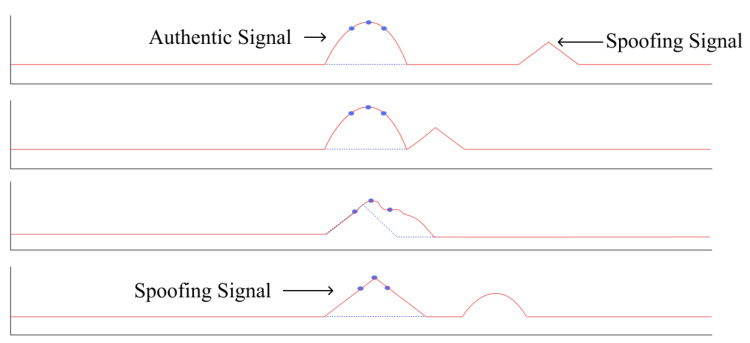
Spoofing attack scheme viewed from the victim receiver. Spoofer: blue dotted lines; sum of spoofer and truth: red solid lines; receiver tracking points: blue dots [53].

**Figure 10:**
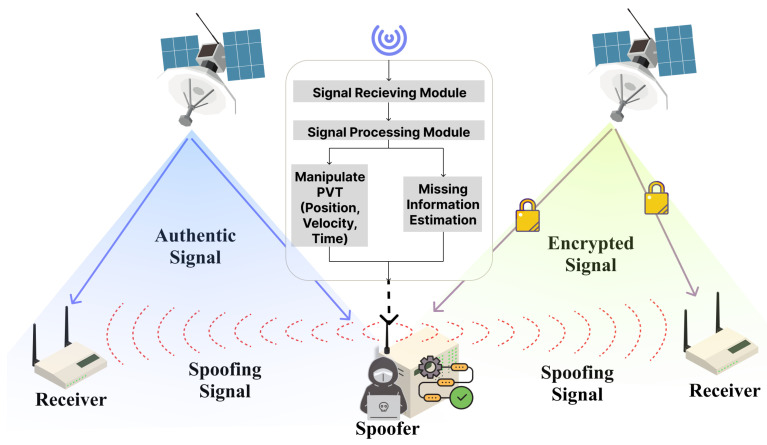
The process of spoofing attacks.

To understand the typical process of a spoofing attack, we can refer to Psiaki’s paper [53]. In Figure 9, five stages of the interaction between the spoofed signal and the true pseudorandom number code autocorrelation function are shown. Initially, in the first stage, the spoofing signal is searching for the true signal’s tracking loop. In the second stage, the real and spoofed signals merge. The third stage shows the spoofed signal being modulated by the code autocorrelation function, and in the fourth stage, the tracking point shifts from the real to the spoofed signal. Finally, in the last stage, the spoofed signal becomes stronger than the real signal and undergoes phase modulation. At this point, the spoofed signal, carrying false positional signals or timing information, is transmitted to the receiver. Such methods of spoofing attacks are conventional. So, to achieve a higher success rate, appropriate techniques, equipment, and new technologies are required. Using the aforementioned principle, SCSs can be spoofed, enabling the execution of various attacks, as illustrated in Figure 10. Therefore, in this section, we will categorize the existing spoofing strategies into three types: signal meaconing spoofing attacks, signal estimation and replay spoofing attacks, and advanced signal modification spoofing attacks.

**Signal Meaconing and Replay Spoofing Attacks:** These attacks are essentially no different from a signal repeater and typically rely on known signal structures [53]. It is a basic form of attack where the incoming signal is delayed and then retransmitted. The primary target of this attack is the code phase time history. For instance, if the manipulated time is 1:59:59.99, the actual time would be 2:00:00.00.

In Wesson’s paper [54], an attack method is presented where an entire block of GNSS RF signals is recorded and played back without needing to separate each signal. This approach does not alter the actual satellite signals’ position, velocity, and time values but introduces the spoofer’s location and velocity to the receiver with an added time delay. In this attack, the spoofer is ideally positioned near the receiver to prevent delays in signal processing, enabling a zero-delay meaconing attack. This allows the spoofer to circumvent the receiver’s defenses against clock drift. Also, the study in [55] demonstrated that the position, velocity, and time values of a receiver can be affected by a spoofing attack through simulations using a station repeater spoofing model.

**Signal Estimation and Replay Spoofing Attacks:** Unlike the previously mentioned attack, in this approach, the spoofer does not have all the information when receiving the signal, but only a part of it [56]. For instance, in the case of military satellites or some civilian satellites with undisclosed security codes, not all information is available to the spoofer, unlike public civilian GNSSs [53]. Therefore, the spoofer needs to estimate the missing information and then introduce a delay to execute the spoofing attack. Typically, estimation and replay attacks are divided into two categories: security code estimation and replay (SCER), and forward estimation attacks (FEAs).

Paper [57] was the first to propose the SCER method, suggesting a technique to deceive receivers through accurate estimation of security codes and deliberate delays. This approach is suitable for various cryptographic techniques, ranging from low-rate methods like navigation message authentication (NMA) to high-rate strategies, including the legacy military GPS Y-code encryption. An FEA is a methodology employed by adversaries to generate counterfeit GNSS signals that are perceived as authentic by the target receiver [58]. Unlike the SCER attack, FEAs are not limited by the immediate estimation of each symbol as it is broadcast. Instead, this approach allows an attacker to produce counterfeit signals that can either delay or precede the genuine signal. This flexibility makes FEAs particularly potent, as it enables the synthesis of signals that closely mimic the authentic ones, thus making the deception more convincing and harder to detect.

**Signal Modification Spoofing Attacks:** Recently, a different form of attack has been introduced, known as the nulling attack. A nulling attack is a specific type of spoofing strategy used against GNSS receivers. In this attack, the spoofer employs nulling signals with amplitudes that are twice those of the corresponding authentic signals. These enhanced nulling signals are activated only during specific data bits, $D_{i}\left(t\right)$, whose polarity the spoofer aims to invert. By doing so, the spoofer manages to induce a false position or timing fix in the target receiver [54]. The detailed process is as follows.

In Equation (2), the first is the counterfeit signal, which works together with the other spoofed signals to create a false location or time reading. The second signal is the inverse of the authentic signal. Therefore, the total number of signals $N_{s}$ is twice the number of authentic signals *N*, denoted as $N_{s}=2N$. It is assumed that the initial *N* signals are the spoofed versions, while the subsequent *N* signals are the nulling versions. The nulling signals are designed to negate the true signals at the receiver, which means for i=1 to *N*, the following conditions are met: $C_{i+N}\left(t\right)=C_{i}\left(t\right)$ and ${\hat{D}}_{i+N}\left(t\right)=D_{i}\left(t\right)$. Moreover, the nulling signals must adhere to $A_{s[i+N]}=A_{i}$, $\tau_{s[i+N]}\left(t\right)=\tau_{i}\left(t\right)$, and $\phi_{s[i+N]}\left(t\right)=\phi_{i}\left(t\right)+\pi$. Signal elimination occurs due to a 180-degree (or π radians) carrier phase shift [56].

The nulling process effectively eliminates any evidence of the genuine signal in the aggregate received signal. Therefore, any anti-spoofing strategies that rely on detecting such duplications are bound to fail. An interesting aspect of the nulling attack is its efficiency in terms of resource utilization. It requires only half as many spoofing channels compared to a general nulling attack, making it a more resource-effective method of spoofing. To successfully execute a nulling attack, the spoofer must be aware of its spatial relation to the victim receiver, as this knowledge is crucial for the precise delivery of the nulling signals.

However, this type of attack is very difficult to successfully execute in the real world and remains largely theoretical. Additionally, there are still no known instances of its practical application [56].

### 4.4. Summary

SCSs face an array of sophisticated attacks that threaten their confidentiality, availability, and integrity, showcasing the critical vulnerabilities inherent in these networks. Eavesdropping, both passive and active, poses a significant risk by enabling unauthorized interception of communications, potentially exposing sensitive personal and national security information. Availability threats, including jamming and DoS/DDoS attacks, disrupt satellite services, affecting everything from military operations to civilian GPS navigation. Spoofing attacks, particularly sophisticated in their execution, compromise the integrity of satellite signals, leading to misleading data being transmitted to receivers. These threats highlight the pressing need for comprehensive security countermeasures that encompass advanced technological defense methods and research to safeguard the vital infrastructure of global communication networks.

## 5. Defense in Satellite Communication Systems

**Figure 11:**
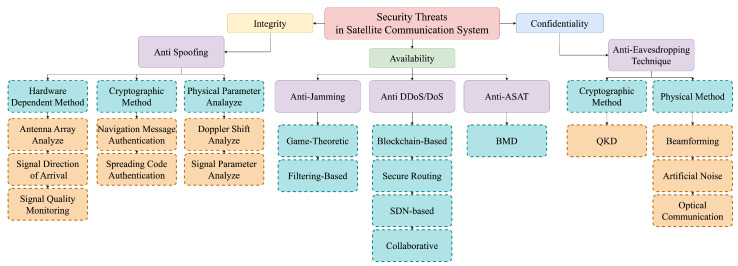
Defense scheme in a satellite communication system.

In this section, we explore countermeasures for the attacks discussed earlier, examining the methods for each attack through a classification criterion. The overall classification is depicted in Figure 11.

### 5.1. Defense Methods for Confidentiality

- Anti-Eavesdropping—Physical methods

**Figure 12:**
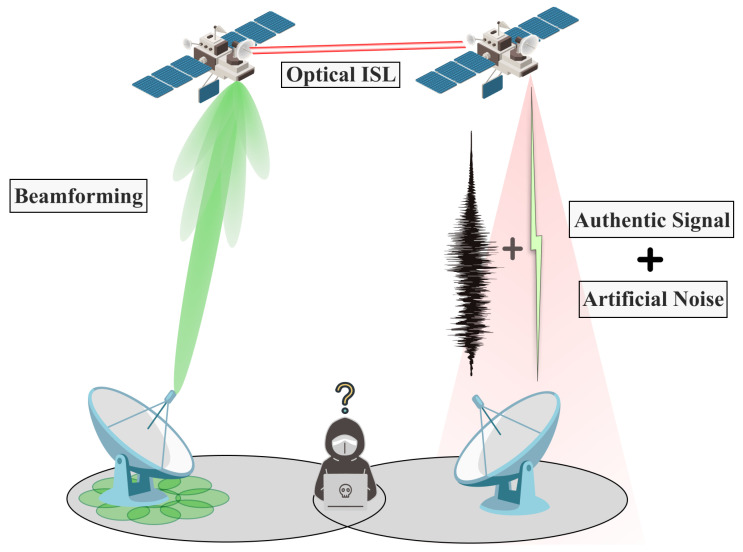
Anti-eavesdropping methods’ scheme.

**Beamforming:** Beamforming is a signal processing technique used in antenna arrays to direct the transmission or reception of signals in specific directions. Beamforming for anti-eavesdropping refers to the use of beamforming techniques in wireless communications to enhance the security against eavesdropping attempts. In most cases, beamforming for anti-eavesdropping in satellites prevents eavesdropping attacks by utilizing the size and directivity of the beam. The overall scheme is shown in Figure 12.

The authors of [59] address the challenge of coexistence between a fixed satellite service (FSS) and cellular networks, specifically in the shared usage of the Ka-band. They present a system model involving satellites and base stations with multiple antennas and FSS terminals and BS users with single antennas, forming a multiple-input single-output (MISO) channel in the millimeter-wave frequency band. To enhance the PLS, they propose two beamforming schemes, non-cooperation-based and cooperation-based schemes, designed to optimize transmission beamforming and protect FSS terminals from eavesdropping. Similarly, ref. [60] presents a model of a MISO wiretap channel, specific to multibeam SCSs, where each ground terminal is associated with a distinct satellite beam, treating interference from other beams as cochannel interference. They concentrate on PHY design for secure SCS, focusing on power allocation and beamforming that is designed to eliminate cochannel interference and nullify eavesdropper signals, with an emphasis on understanding the impact of the eavesdropper’s channel condition on security system design. Zheng et al. [61] introduce a novel framework for PLS in broadband multibeam satellite systems, emphasizing new beamforming requirements. It considers a general eavesdropping scenario, where each legitimate user could be targeted by multiple eavesdroppers, either unique to them or shared across users. Also, they propose a partial zero-forcing approach, designed to make the signal intended for legitimate users orthogonal to the channels of eavesdroppers. To optimize transmit beamforming, an iterative algorithm based on semi-definite programming relaxation is developed.

**Adding Artificial Noise:** Artificial noise (AN) for satellite anti-eavesdropping is a strategy used in SCSs to enhance security against unauthorized interception. The noise is designed to overlap with the eavesdropper’s signal space, making it difficult for them to distinguish the actual communication from the noise. This process is depicted in Figure 12.

For example, ref. [62] introduces a novel deep neural network (DNN)-based secure precoding scheme, named the deep AN scheme, for use in MISO wiretap channels. This scheme utilizes a DNN for the joint design and optimization of precoders for both the information signal and the AN signal. It inputs the estimated channel of the receiver and outputs predicted precoding vectors for both the information and AN signals. Ding et al. [63] propose the artificial-noise-aided two-way opportunistic relay selection scheme, a novel approach to enhance security in wireless relay networks. In this scheme, two sources, S1 and S2, transmit signals to relays in separate time slots, with each source simultaneously emitting AN during the other’s transmission to protect against eavesdropping. This scheme also incorporates a defense against side lobe eavesdropping, where the AN is tailored to interfere with potential eavesdroppers by introducing perturbations in their received signal. However, the effectiveness of this perturbation can be impacted by errors in channel state information estimation.

**Optical Communication:** Optical wireless communication in satellites represents a technology that uses light, typically in the form of lasers, to transmit data wirelessly. This offers unique advantages for anti-eavesdropping measures compared to traditional RF. This process is depicted in Figure 12.

Pradhan et al. [64] propose a line-of-sight, non-diffused link setup for inter-satellite optical wireless communication systems, employing coherent optical quadrature phase-shift keying modulation. This system is tailored for high bit-rates of up to 400 Gbps and is capable of significant coverage across various orbits, including LEO, MEO, and GEO. Also, a high-speed inter-satellite optical wireless communication system was designed in [35]. The research on free-space optical communication make it inherently more secure against eavesdropping compared to traditional RF communication. The narrow, direct path of the laser beam and its inability to penetrate walls create a communication channel that is hard to intercept without being noticed, thereby enhancing security and privacy in optical communications, especially in space, where line-of-sight communication paths are common [65].

b. Anti-Eavesdropping—Cryptographic Methods

**Quantum Key Distribution:** QKD is a technology that transmits secret keys using quantum principles over a communication network. Recently, with the rapid advancement of quantum computer research and the development in various countries, the security of contemporary cryptography based on mathematical complexity has started to be significantly threatened. As a response, QKD technology, which derives its security not from computational difficulty but from physical laws, has emerged. Recently, there has been development in the key distribution technology based on SCSs [66].

**Figure 13:**
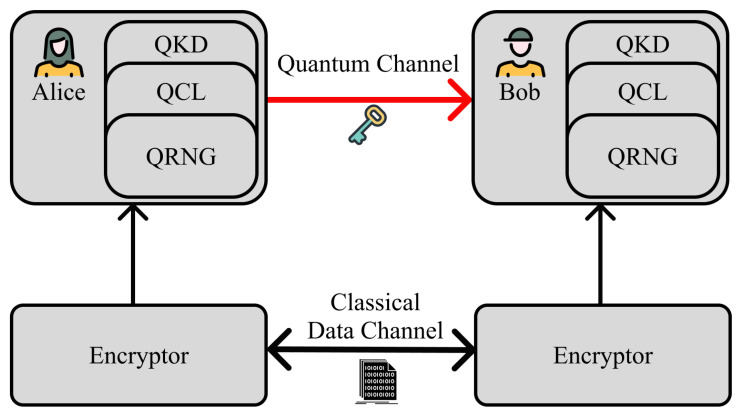
Quantum key distribution scheme.

The process of QKD is depicted in Figure 13 and involves the following steps. First, Alice sends photons to Bob, encoding bits (0 or 1) onto each photon’s quantum properties, such as the polarization direction. The photons travel from Alice to Bob through a quantum channel, which could be fiber optic cables or free space. Second, Bob measures the incoming photons using a basis that he chooses randomly. This basis may or may not match the basis used by Alice for encoding. After the transmission, Alice and Bob publicly share which basis they used for each photon. They discard any bits where their bases do not match. Also, they compare a subset of their matching bits to check for errors or eavesdropping. A high error rate indicates a potential eavesdropper. Lastly, the remaining matching bits, where the bases align and no errors are found, form the secret key.

QKD has the following features: (1) Immunity to mathematical decryption. Quantum cryptography is based on irreversible physical natural phenomena, making it immune to decryption through mathematical approaches. (2) Resistance to replay attacks. Quantum cryptography is immune to replay attacks. Any slight mistake by an attacker in measuring the signal distorts it, leading to the receiver detecting that the signal has been intercepted. (3) Principle of non-clonability. In quantum cryptography, a photon cannot be perfectly copied due to the principle of non-clonability, a fundamental phenomenon in quantum mechanics. Without perfect measurement of the original signal, distortion occurs, making it impossible to obtain information [67].

**Figure 14:**
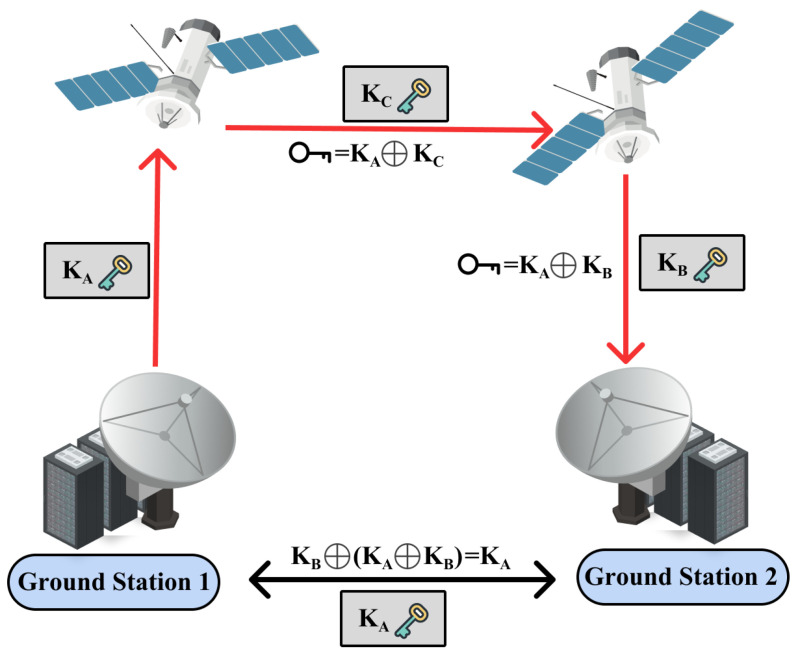
Key exchange process in QKD.

Because of these features, QKD is considered the most perfect solution against eavesdropping and is one of the ideal methods for generating one-time pads. The process is depicted in Figure 14. Like Figure 14, numerous studies have been conducted on using this technology in SCSs. Early research [68] discovered the ability to create entanglement in the Earth’s orbit. Subsequently, we progressed rapidly to stages where actual data could be sent. Initially, satellite-based QKD was close to the experimental stage, resulting in a trade-off between key delivery speed and distance [69,70,71,72]. Later, satellite-based QKD evolved to capture both distances and key transmission speeds [73,74], and research also emerged that could achieve complete security purely through entanglement without the need for a trust relay [75]. Moreover, research incorporating various technologies like twin fields and technologies that are capable of much longer distances has also been conducted [76].

### 5.2. Solutions for Availability

- Anti-Jamming

Anti-jamming in SCSs refers to the techniques and strategies used to protect satellite signals from intentional interference or jamming attacks. These methods aim to ensure the integrity and availability of SCSs by mitigating the effects of jamming. Anti-jamming can involve a variety of approaches, such as using spread spectrum technologies, frequency hopping, signal encryption, and implementing robust error-correcting codes. These techniques enhance the resilience of SCSs against deliberate attempts to disrupt or degrade the signal quality. An overall summary of anti-jamming and its pros and cons is shown in Table 4.

**Game Theoretic Approaches:** Mitigating a jamming attack using a game-theoretic approach in SCSs involves formulating the interaction between a jammer (attacker) and a communication system (defender) as a strategic game. In this framework, each participant is considered a player with specific strategies. The goal is to analyze and predict the behaviors of the jammer and the defender, with the aim of finding optimal strategies for the defender to minimize the impact of jamming.

Wang et al. [77] apply game theory to address FHSS satellite jamming. They formulate the minimization of the damaging effect of satellite jamming attacks as a two-player asymmetric zero-sum game framework, where the payoff is modeled as the channel capacity of the defender under white additive Gaussian noise. Also, ref. [78] presents a two-stage anti-jamming scheme for satellite internet communication. This scheme integrates a deep-reinforcement-learning-based routing algorithm with a fast response anti-jamming algorithm, using Stackelberg game theory for dynamic and uncertain environments.

**Filtering-based Approaches:** Mitigating a jamming attack using a filter-based approach in SCSs involves implementing specific filtering techniques to isolate and remove the jamming signal from legitimate communication signals. This approach typically uses advanced signal processing algorithms to distinguish between the noise introduced by the jammer and the genuine satellite signals.

The authors of [79] discuss a range of anti-jamming strategies for small satellites. They cover modulation adjustments, wave filtering, spread spectrum techniques, smart antenna technology, and methods to counter broadband interference, emphasizing an integrated approach for enhanced system robustness against jamming. Similarly, ref. [39] also introduces an anti-jamming technique that is a dual-filter approach combining the augmented-state approximate conditional mean filter and discrete wavelet transform filter to mitigate FM jamming in GPS receivers. This method is effective for both single and multiple FM jammers, with simulations demonstrating its efficacy, especially in high jammer-to-signal scenarios.

b. DoS/DDoS Mitigation

DoS and DDoS attacks aim to neutralize or halt the function of devices. Typically, these types of attacks can be countered in various ways, such as detecting intrusions in the network or verifying changes. In SCS networks, defending against DoS and DDoS attacks usually involves ground-satellite integrated systems and detection from the ground, as computational costs are high in satellite systems. Therefore, this solution is approached by dividing it into four categories: blockchain-based solutions, secure routing-based solutions, SDN-based solutions, and collaborative-based solutions. An overall summary of DoS and DDoS attack mitigation and its pros and cons is shown in Table 5.

**Blockchain-based Mitigation:** Blockchain technology has recently emerged and allows for transparent information sharing within a network. Typically, a blockchain database stores data in sequentially linked blocks, making them detectable by other users if any chain is altered or deleted without network consensus. This ensures the blocks in a blockchain maintain temporal consistency, guaranteeing the immutability and unchangeability of transactions [11]. In SCSs, these features are used to enhance the stability and availability of each node, offering solutions for mitigating attacks like DDoS attacks.

Feng et al. [80] developed a new security framework for mobile SCS networks (MSNETs) using blockchain technology. This framework addresses security challenges in MSNETs by integrating blockchain with a delay-tolerance network (DTN) architecture and satellite constellation management algorithms. The blockchain method is employed via two key methods: securing data communication within the DTN structure and combining it with satellite constellation management to defend against physical cyber attacks. This approach ensures not only the detection of cyber attacks such as flood attacks but also enhances defense capabilities through improved communication and satellite management. Similarly, ref. [81] introduces a new security architecture for SCS networks using blockchain technology. This approach integrates satellite and ground equipment, with a ground base station processing and recording information in a distributed blockchain to eliminate malicious nodes. The architecture includes key features such as enhanced access control, confidentiality, public key authentication for message source verification, and a decentralized system for data management and authority. Additionally, it manages IoT device communications and control commands, significantly improving communication security and protection against unauthorized access like DDoS attacks, data theft, and hacking. Ref. [82] proposes an access control and intrusion detection framework, named ACID, for blockchain-based SCSs. This framework introduces a token-based access control mechanism for smart contracts, allowing only authorized users to trigger specific smart contracts and preventing illegal access. Additionally, it incorporates an intrusion detection mechanism capable of effectively detecting malicious attacks on smart contracts.

**Secure-Routing-based Mitigation:** Routing and security play crucial roles in wireless networks. Specifically, since routing is fundamental in wireless communication, developing efficient routing strategies is a key element [83]. Recent studies [45,84,85] have highlighted attacks on such routing strategies, necessitating protective measures. In SCSs, the limited computational resources and energy efficiency of satellites significantly impact routing, leading to the development of methods that can enhance the routing security. Li et al. [86] focus on the secure LEO trust-based (SLT) algorithm, designed to secure routing in LEO satellite networks against internal malicious attacks. They utilize a distributed trust evaluation model based on Dempster–Shafer evidence theory, eliminating the need for a centralized infrastructure. This model calculates direct, indirect, and aggregate trust values among satellites to assess their trustworthiness. The SLT algorithm can be integrated with the Orbit Prediction Shortest Path First (OPSPF) [87] routing protocol, enhancing network security by detecting and isolating malicious nodes, thereby improving packet delivery rates and reducing packet loss. The algorithm shows increased effectiveness in networks with a higher presence of malicious nodes, indicating its robustness and adaptability in challenging environments.

**SDN-Based Mitigation:** An SDN is an approach to network virtualization and containerization that optimizes network resources and facilitates rapid adoption of new methods for applications and traffic in changing network environments. The integration of SDNs into SCS networks is a recent transformative development, enhancing the flexibility and functionality of these networks. This is particularly evident in two recent proposals focusing on the application of SDNs in space-based networks, addressing key issues like DDoS attack detection and energy-efficient network designs.

First, ref. [88] combines an optimized long short-term memory (LSTM) DL model with a support vector machine (SVM), specifically tailored for the SDN architecture. This approach directly addresses the vulnerability of traditional SDN controllers to network attacks, which can risk network paralysis. By utilizing time series data and flow characteristics, the method aims for accurate detection of DDoS attacks, thereby reducing false alarms and refining the overall detection process. The effectiveness of this method is underscored by experimental results, demonstrating high accuracy and low false alarm rates, thus offering a reliable solution for enhancing the security of space-based networks against such threats. Also, the author of [89] presents an innovative approach that not only mitigates DDoS attacks but also prioritizes energy efficiency. They introduce an energy consumption model and an improved network topology algorithm aimed at minimizing energy usage, a critical consideration given the limited resources and high vulnerability to DDoS attacks in satellite networks. Furthermore, this proposal includes a DDoS mitigation mechanism employing deep reinforcement learning algorithms, adept at managing abnormal traffic and reducing the extra energy consumption that typically results from processing such traffic in satellite nodes. The combination of these approaches in the software-defined satellite network context is proven to effectively decrease energy consumption while simultaneously bolstering network security, thereby making software-defined satellite networks more resilient to DDoS threats and more energy efficient.

**Collaborative-Based Mitigation:** Collaborative defense in satellite networks refers to a strategy where multiple terrestrial or satellite nodes or systems work together to enhance network security and resilience. This approach often involves sharing information and resources among satellites or ground stations to identify and mitigate cyber attacks, including DDoS attacks. The collaboration can be based on advanced technologies such as blockchain for secure data sharing or artificial intelligence (AI)-driven algorithms for threat detection. This synergy allows for a more robust and adaptive defense mechanism, crucial for the unique challenges faced by satellite networks in space.

Guo et al. [90] present the Distributed Collaborative Entrance Defense (DCED) framework to protect the satellite internet from DDoS attacks, addressing its unique vulnerabilities like the limited computing power and bandwidth. DCED includes a two-stage detection digesting process for traffic feature extraction. First, the entrance traffic digest (ETD) records six items of traffic features using the destination IP addresses as an index. Second, the defense plane digest (DPD) cross-detects different types of DDoS attacks from several perspectives using information theory, enabling the handling of various attacks like UDP, SYN, ACK, and ICMP attacks. Also, smart-contract-based virtual aggregation of digests with blockchain and MapReduce is utilized. Hajizadeh et al. [91] introduce a secure, distributed model for cyber threat intelligence (CTI) sharing in SDNs using blockchain technology, specifically Hyperledger Fabric, to provide a secure and tamper-proof environment. This model is integrated with an SDN to enhance the defense against cyber threats like zero-day attacks and malware, ensuring fast enforcement of security policies based on shared CTI. Also, it addresses CTI sharing challenges such as security, scalability, and trust and demonstrates its effectiveness in mitigating DDoS attacks within multi-domain SDNs. The platform maintains its network security and participant privacy through authenticated access and smart contracts for reliable service-level agreements.

c. Anti-Energy Attack

**Figure 15:**
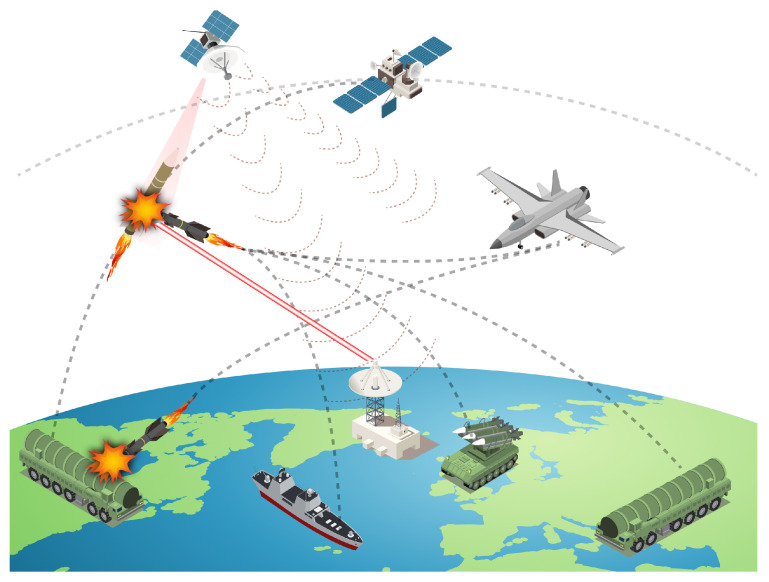
Illustration of ASAT defense methods.

In the previous section, we highlight that energy attacks on satellites are predominantly direct attacks using missiles. This implies that missile defense systems could potentially be used for defense. Specifically, BMD systems might be suitable for countering ASAT weapons. This is because intercepting ASAT weapons requires reaching altitudes where the satellites operate, and the nature of ballistic missiles is not significantly different from the components of ASAT weapons [92]. Therefore, in this section, we will examine BMD systems. The overall illustration is shown in Figure 15.

In ballistic missile defense, there are three critical phases of interception. The boost phase involves intercepting the missile shortly after its launch while the engines are still active. The mid-course phase targets the missile when it is in space during the period of its trajectory where it is not powered and is in free flight. Finally, the terminal phase occurs when the missile re-enters the Earth’s atmosphere and is nearing its target, which is the last opportunity for interception. Given that ASAT threats would have already intercepted satellites during the mid-course or terminal phases, our focus should be solely on systems capable of intercepting these threats during the boost phase.

In the context of boost phase missile defense, “early intercepts are best”. Destroying a ballistic missile during its boost phase is considered the optimal approach in missile defense as it prevents the missile from deploying countermeasures and stops the warhead from gaining the velocity needed to reach its target [93]. However, intercepting a missile during this phase, while it is still ascending and fighting against Earth’s gravity, presents significant challenges. Firstly, the boost phase lasts for only a brief period, requiring rapid detection and transmission of precise information about the missile’s launch. Secondly, to successfully intercept the missile during this phase, the defensive interceptor must either be positioned very close to the launch site or be capable of extremely high speeds to catch up with the accelerating missile. Current technological advancements are playing a significant role in potentially making boost phase defense more viable. Innovations in sensor technology, like gallium-nitride-based radar, could reduce the timeline for detecting a boosting ballistic missile. Furthermore, advancements in infrared sensor performance and image processing, as well as the declining costs of satellite manufacturing and launch, are also contributing to the feasibility of boost-phase defense. Directed energy technology, particularly lasers, is another area being explored for boost-phase defense.

Russia has developed a laser platform called the Beriev A-60 for anti-ballistic missile purposes, a project that dates back to the Soviet era. Furthermore, Russia has been a forerunner in laser weapon technology, reportedly deploying laser systems for missile interception purposes in March 2018. This deployment indicates Russia’s significant advancements in directed energy weapons, particularly in the context of missile defense [94].

The United States has developed a high-energy-laser-based weapon system called the High-Energy Liquid Laser Area Defense System (HELLADS). This system is designed to defend against surface-to-air threats such as rockets, artillery, mortars, and surface-to-air missiles. The HELLADS program aims to create a 150-kilowatt laser system that can counter these threats effectively. The core aspect of this system is its miniaturization, allowing it to be mounted on aircraft for the purpose of intercepting ballistic missiles during their boost phase [46].

### 5.3. Solutions for Integrity

Anti-Spoofing

Anti-spoofing refers to the range of techniques and strategies used to protect GNSSs in SCSs from spoofing attacks. The main goal of spoofing detection is to distinguish between the spoofing signal and the actual satellite signal in the received signal. If the real signal and the spoofing signal are not successfully detected, as seen in Section 3, this can have serious implications, especially in critical SCS parts such as the GNSS.

**Figure 16:**
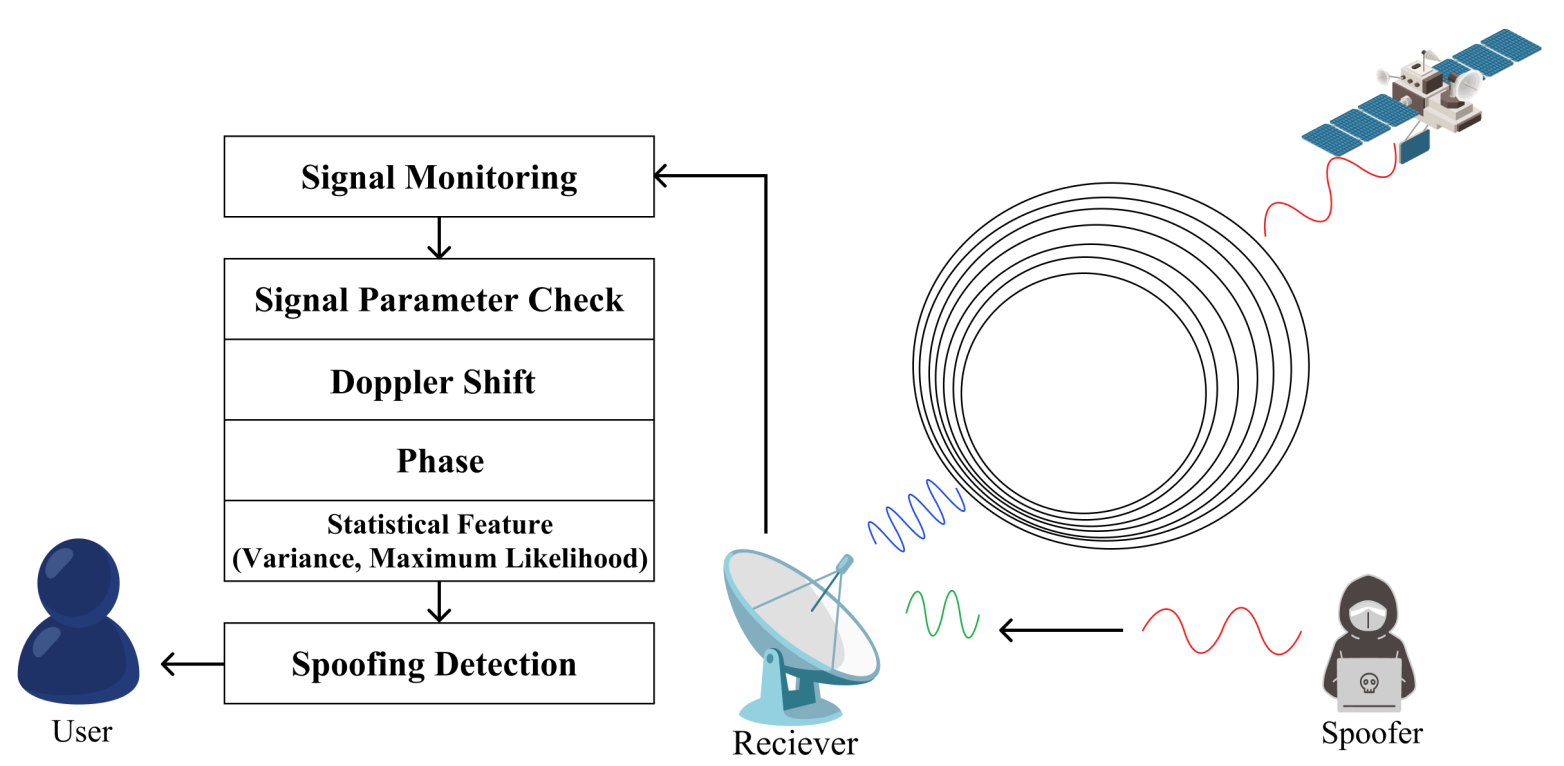
Anti-spoofing using physical parameter analysis.

**Figure 17:**
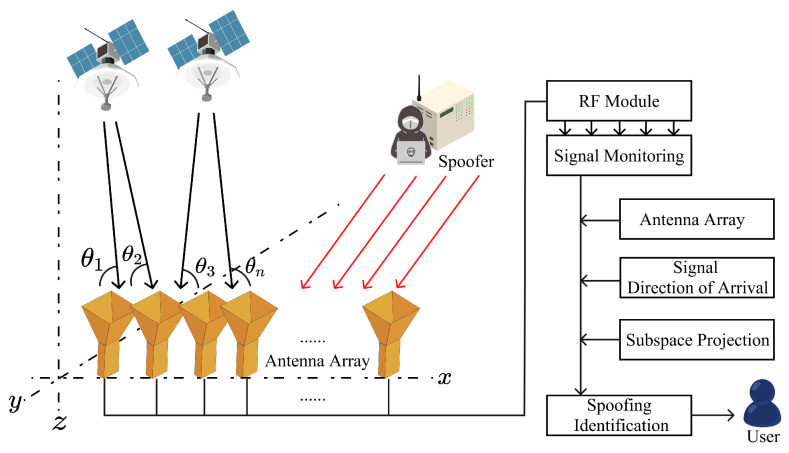
Anti-spoofing using hardware-dependent parameter analysis methods.

So, in this section, we categorize anti-spoofing methods into three main types: physical parameter analysis methods, hardware-dependent methods, and cryptographic methods. Under the physical parameter analysis method section, we will look at approaches like Doppler shifts, signal parameter statistics analysis, arrival time and time difference, and residual signal detection. The overall steps of this method are shown in Figure 16. In the hardware-dependent parameter analysis method section, we will explore antenna arrays, angles of arrival, subspace projection, and signal quality monitoring. And the overall steps of hardware-dependent method are shown in Figure 17. Finally, for the cryptographic methods, we will examine NMA, protocol-based NMA, spreading code authentication (SCA), and encryption.

#### 5.3.1. Physical Parameter Analysis Methods

(i) Doppler Shift Analysis: The Doppler shift, also known as the Doppler effect, is a change in the frequency of a wave in relation to an observer who is moving relative to the wave source. In SCSs, the satellite usually moves at a high speed relative to the receiver, leading to the occurrence of the Doppler shift. This is observed as a change in the frequency of a wave in relation to an observer moving relative to the wave source. In this context, if the Doppler shift value transmitted from the actual satellite and the Doppler shift value of the received signal are consistent, it is considered a normal signal. However, if the received Doppler shift value exceeds a certain predefined limit, it may be indicative of a spoofing attack. The Doppler effect’s role in combating GNSS spoofing attacks is highlighted in various research studies, each proposing unique methods to utilize this phenomenon. Paper [95] leverages Doppler offset detection (DOD) to differentiate between authentic and fake signals, relying on a model that highlights the necessity for a high level of resources for spoofers to match the Doppler offset of multiple satellite signals. In contrast, ref. [96] focuses on the Doppler effect’s frequency changes due to the relative movement between the wave source and observer, especially in GNSS satellites. This study notes that spoofers, usually at a lower altitude, produce a larger Doppler shift, aiding in distinguishing between real and spoofed signals. Furthermore, the authors of [97] discuss the proportional relationship between the Doppler frequency and relative speed and its inverse proportionality to wavelength, leading to significant carrier frequency offsets in GNSS satellites. This paper emphasizes how, for static receivers, the Doppler shift changes slightly, whereas for moving receivers, these changes are more noticeable. This difference in Doppler shift changes between static and moving receivers helps in identifying authentic signals, as spoofed signals from a single spoofer will show uniform shift changes. Lastly, ref. [98] describes how the observed wave frequency differs from the transmitted frequency due to the relative velocity between the transmitter and receiver. This study advocates for monitoring the Doppler shifts of various GPS satellites to detect spoofing, as spoofed signals from the same platform exhibit identical Doppler shifts, unlike the varied shifts of authentic signals.

(ii) Signal Parameter Analysis: GNSS spoofing detection using signal parameter analyses can be conducted in various ways, each utilizing different aspects of statistical signal analysis to enhance the accuracy and reliability of spoofing identification. In [99], phase-only analysis of variance (PANOVA) tests are introduced, focusing on detecting differences in the phases of sample groups to identify spoofed GNSS signals. This method effectively characterizes the spatial signature of GNSS signals, taking advantage of the difficulty experienced by spoofers when replicating certain signal features like correlation shapes and phase spatial signatures. In another approach, ref. [100] analyzes the sum-of-squares detector. This detector uses carrier phase measurements from two spatially separated GNSS receivers, formulating a decision statistic for real-time applications. The effectiveness of this method was proven through simulations and real GPS data experiments, demonstrating its practicality in revealing spoofing attacks. Ref. [101] proposes a new method based on the sequential probability ratio test (SPRT), emphasizing GNSS signals’ vulnerability to jamming and spoofing. This method, suitable for the acquisition stage, compares various techniques for spoofing detection, including the correlation between two receivers and multi-antenna processing, acknowledging some practical limitations for civilian GNSS receivers. Ref. [102] extends the power-distortion (PD) detector, focusing on monitoring received power and correlation-profile distortions to detect GNSS carry-off-type spoofing, jamming, or multipath interference. The improved PD maximum-likelihood (PD-ML) detector enhances the classification accuracy, differentiating spoofing from jamming and multipath-affected data. Finally, ref. [103] proposes a novel technique using differential code phase for the time difference of arrival (TDOA) estimation of spoofing signals. This method marks a significant shift from conventional cross-correlation-based TDOA estimation techniques, highlighting the importance of TDOA in localizing GNSS spoofing signals.

#### 5.3.2. Hardware-Dependent Parameter Analysis Methods

(i) Antenna Array Analysis: The role of antenna arrays in anti-spoofing and jamming resistance for GNSS receivers is critical, as highlighted in various studies. These arrays, comprising multiple closely spaced antennas, can either form a fixed receive beam width or adaptively modify it, enhancing the resistance to jamming. In [104], NavAtel’s GAJT uses a seven-element controlled reception pattern, and QinetiQ employs a null-forming algorithm, both recognized for their jamming resistance. However, implementing adaptive modification, usually in safety-critical applications, can be complex and costly. One challenge is that measurements in receivers with an antenna array and beamforming equipment can be distorted due to time processing, affecting high-accuracy applications like carrier phase measurements. In another approach, in ref. [105], the focus shifts to double-antenna systems, which are particularly useful in scenarios where antennas are not synchronized. While synchronizing measurements typically require two receivers connected to a common oscillator or precise timestamped measurements, this can be impractical or unreliable in low-cost receivers or spoofing attacks. To overcome synchronization challenges, this study proposes a detection method based on double-antenna power measurements using the correlation outputs of the code-tracking loop for spoofing detection, suitable for typical anti-spoofing applications [106,107]. Both approaches assume that spoofing signals originate from a single point source and employ spatial domain processing techniques to isolate and discard these signals. This is implemented before the despreading and acquisition stages in a GNSS receiver, significantly reducing the computational load by eliminating the need for extensive searching in the code and Doppler domains to differentiate between authentic and spoofing signals. Also, the system models assume an arbitrary configuration of an N-element antenna array, where the distance between adjacent elements is less than half of a GNSS signal carrier wavelength. Also, they use the same in their experimental setups. This describes a complex baseband representation of received signals at the array, considering both authentic and spoofing signals involving steering vector matrices and noise vectors.

(ii) Direction of Arrival: The principle of direction-of-arrival (DOA) sensing is a pivotal element mitigation method against GNSS spoofing. In [108], a system developed in Psiaki’s lab is discussed, which utilizes interferometry principles for spoofing detection. This system employs software and two antennas to measure the carrier phase, a property critical in discerning signal characteristic variations between antennas. This variation is instrumental in determining the signal’s angle of arrival. By analyzing these angles, the system can identify inconsistencies indicative of spoofing based on the fact that while a spoofer transmits false signals from a single antenna, authentic GPS signals originate from multiple satellites and thus arrive from different directions. Similarly, ref. [109] focuses on a novel spoofing detection technique that relies on DOA estimation. This approach involves categorizing emitters and selecting those with high elevation angles. DOA estimation is crucial for distinguishing between authentic and spoofing signals, especially when they emanate from different spatial directions. The technique’s reliance on the DOA allows for more precise identification of spoofed signals by contrasting the spatial characteristics of authentic and counterfeit signals.

(iii) Subspace Projection: Subspace projection introduces versatility to techniques for GNSS security. Whether it is adapting to dynamic jamming characteristics or efficiently mitigating spoofing attacks in single-antenna systems, subspace projection provides a method for enhancing the reliability and security of GNSS receivers against sophisticated interference threats. Ref. [110] highlights a significant enhancement in subspace projection for GNSS spoofing and jamming mitigation. Traditional subspace projection methods, while effective, face challenges regarding inaccuracies in instantaneous frequency estimates, particularly when facing attackers with rapidly changing frequency characteristics. To counter this, this paper introduces an adaptive-partitioned subspace projection technique. This approach divides the received data vector into adaptive block sizes based on the attacker’s chirp rate estimates, accommodating the varying IF characteristics. The signal is then projected onto the attacker’s orthogonal subspace constructed from these IF estimates. This technique’s adaptability to the jammer’s sweep rate, by adjusting the time-frequency observation window length, is key. This adaptability ensures consistent instantaneous phase estimation errors, providing an effective solution against frequency-varied jamming. Similarly, ref. [111] presents a robust spoofing mitigation algorithm using subspace projection tailored for single-antenna GNSS receivers. This approach uses the subspace projection method to effectively eliminate spoofing signals from the received signal by projecting them onto the orthogonal null space of the spoofing signals. This process involves approximating the subspace of spoofing signals and subtracting this estimation from the input signals to isolate the authentic signals and ambient noise. This method is particularly valuable for its independence from the number of antennas, making it a versatile solution for a wide range of GNSS receivers.

(iiii) Signal Quality Monitoring: Signal quality monitoring (SQM) techniques detect anomalies or distortions in the received GNSS signals using specific algorithms to specify interference, jamming, or spoofing attacks. Ref. [112] introduces an evolved version of signal quality monitoring for spoofing detection that assesses the quality of the correlation function using two metrics. This includes a ratio test and extra correlators for detecting asymmetries in the correlation function, effectively differentiating between multipath and spoofing interference. The algorithm’s capability to detect spoofers with varying code delays is emphasized, necessitating the joint detection of both metrics to distinguish between multipath interference and spoofer attacks. Another study [113] presents the SQM technique, a low-complexity algorithm designed for anti-spoofing. The SQM technique employs ratio test metrics to detect anomalies in the correlation peak and the presence of vestigial signals, using pairs of correlators and extra-correlators to monitor distortions. This technique includes a calibration phase for establishing baseline values and a detection window for computing thresholds to declare the presence of a spoofer. Its effectiveness is validated in the Texas spoofing test battery, known as TEXBAT scenarios, showing notable success in identifying matched-power spoofing attacks under static and dynamic conditions. Similar to both these studies, ref. [114] focuses on Galileo signals and seeks to detect abnormally shaped or asymmetric correlation peaks caused by the interaction between authentic and counterfeit signals. It underlines the severe risks posed by spoofing attacks, which can mislead GNSS receivers, potentially leading to adverse outcomes. This paper utilizes additional correlator branches for spoofing detection, assessing the correlation peak’s distortion due to spoofing attacks. Various spoofing scenarios, including changes in signal parameters like delays, Doppler shifts, and amplitudes, are analyzed, with different SQM metrics evaluated for their effectiveness in detecting spoofing attacks under these diverse conditions.

#### 5.3.3. Cryptographic Methods

(i) Navigation Message Authentication: NMA is an anti-spoofing method for GNSSs. It involves verifying the integrity and authenticity of the messages sent by GNSS satellites to receivers. NMA is a method to ensure that the navigation data received by a GNSS receiver are genuine and have not been tampered with or fabricated by a spoofer.

Ref. [115] discusses an anti-spoofing methodology using QZSS L1C/A and L1-SAIF signals, highlighting how signature data generated from a part of the L1C/A navigation message are broadcasted for authentication. This study emphasizes the vulnerability of civilian GPS signals, traditionally not intended for security-critical applications, to various forms of interference and attacks. It also reviews different methodologies to combat spoofing and meaconing problems, detailing the use of QZSS L1-SAIF signals for broadcasting signature data for signal authentication. This paper explains the process of spoofing GPS/QZSS receivers and the manipulation of receiver output data. Also, ref. [54] presents a practical technique for authenticating civil GPS signals, combining the cryptographic authentication of the GPS navigation message with signal timing authentication. This approach aims to secure civil GPS receivers against spoofing attacks and discusses NMA as a basis for civil GPS signal authentication, where the navigation message is encrypted or digitally signed for verification by the receiver. It provides a generalized model for security-enhanced GNSS signals and describes various spoofing attacks, including meaconing and SCER attacks. The authors of [116] discuss an anti-spoofing scheme for BeiDou-II NMA and spread spectrum information (BD-II NMA&SSI). This scheme is designed to protect BeiDou II D2 navigation messages from spoofing attacks using cryptographic algorithms and spread spectrum information. Unlike the previous studies, ref. [117] presents a new type of method that uses the timed efficient stream loss-tolerant authentication (TESLA) protocol for NMA in GNSSs. TESLA, originally designed for internet applications, has been adapted for GNSSs to address their unique requirements. The key features of TESLA in this context include the use of symmetric cryptography, which lowers the receiver’s computational burden, and the generation of a one-way key chain by the sender, with keys disclosed in reverse order. Users verify each key using a previously authenticated key or the root key, which is typically authenticated using asymmetric encryption techniques like digital signatures. To suit GNSSs, TESLA underwent modifications, such as reducing the key size and bandwidth through truncating key chain outputs and introducing deterministic padding, which may incorporate elements like the Galileo system time.

(ii) Spreading Code Authentication: SCA is a technique in anti-spoofing that focuses on securing the GNSSs used in satellite signals, which are essential for accurate positioning and timing information. SCA works by ensuring that the spreading codes received by a GNSS receiver are authentic and have not been manipulated or imitated by a spoofer.

For example, the authors of [118] discuss the generation and application of supersonic codes for GNSSs. The key idea is multiplexing supersonic codes with an open code, achieved through block-cipher encryption of the open code. This process generates an encrypted code, referred to as the “fundamental code”, valid for a predetermined crypto-period. Supersonic codes, transmitted alongside open codes like GPS C/A or Galileo OS, are based on symmetric cryptographic schemes and block ciphers. This setup enables direct authentication without time dependency and allows interoperability with open services. Also, Anderson et al. [119] propose a method named Chimera to enhance the security of GPS civilian signals against spoofing attacks. The Chimera concept involves authenticating both the navigation data and the spreading code of a GPS signal through ‘time binding’. In this process, the spreading code is punctured by markers cryptographically generated using a key derived from the digitally signed navigation message. This integration of the navigation message and the spreading code into a single, inseparable signal makes it challenging for spoofers to replicate the correct signal.

### 5.4. Summary

In addressing the diverse threats to SCSs, a range of countermeasures have been developed to safeguard confidentiality, availability, and integrity. For confidentiality, techniques such as beamforming, adding artificial noise, and leveraging optical communication enhance the security against eavesdropping. Cryptographic methods, including quantum key distribution, provide a foundational layer for secure communications. To ensure availability, anti-jamming strategies employ game-theoretic- and filtering-based approaches, while DoS/DDoS mitigation leverages blockchain technology, secure routing, SDN-based solutions, and collaborative defenses to protect the network infrastructure. For integrity, anti-spoofing measures analyze physical parameters, utilize hardware-dependent analysis, and apply cryptographic techniques to detect and mitigate spoofing attacks.

As a result, the field of research on attacks and defenses is constantly advancing, driven by technological advancements and the evolving paradigm of communication systems. In addition, international politics and policies often dictate the SCS research direction, particularly on security attacks and defense. Therefore, it is important to consider international policies and strategies and keep up with communication technology trends.

## 6. Policies and Strategies in Satellite Communication

The role of SCSs has expanded dramatically, now encompassing a wide range of applications from military operations to civilian real-time communication and location tracking. With their increased usage, the vulnerability of these systems to various threats has also escalated significantly, as evidenced by rising concerns over cyber attacks and the development of anti-satellite (ASAT) technologies [120,121]. The ongoing geopolitical tensions, notably between China and the United States, have intensified the strategic importance of space assets, pushing these confrontations towards cyber warfare and leading to potential breaches of international agreements like the Outer Space Treaty [122,123].

In response to these threats, nations are crafting diverse space security doctrines, strategies, and policies. These range from offensive strategies that deploy ASAT weapons to disable satellite systems, potentially crippling their functionality [124], to defensive strategies aimed at bolstering the resilience of SCSs against a spectrum of threats including cyber and physical attacks, space debris, and natural phenomena [125]. This resilience is crucial for ensuring continued operation despite threats, given the challenges of maintenance in space. Therefore, various countries are focusing on strategies and policies that either enhance resilience in SCSs or threaten it.

Also, the development and implementation of these national policies and strategies are pivotal in defining the security landscape of SCSs. Given the critical role of SCSs as national assets, it is essential to include a detailed analysis of these policies and strategies. Firstly, this provides an overall understanding of how legal and strategic frameworks impact technical decisions and implementations. Secondly, as SCSs are governed by strict international and national laws, reviewing policies helps to ensure that technical designs and operations comply with these regulatory standards. Furthermore, understanding geopolitical dynamics and defensive strategies enhances the security posture of SCSs, enabling effective threat mitigation and defense against a range of challenges, including cyber and physical attacks. Lastly, this inclusion offers insights into future technological and regulatory trends, fostering proactive planning. Consequently, this analysis not only helps in understanding the current state of SCS security but also informs future efforts to enhance system robustness and overall security. Therefore, in this section, we aim to clarify these critical aspects and highlight their significant impact on global satellite communication.

**The United States of America:** The United States’ space security strategy regarding SCSs and space assets has always maintained a competitive stance. While the U.S. has consistently demonstrated technological superiority from the past to the present, there is an ongoing effort to further enhance competitiveness due to the global leveling up of space technology. Additionally, the U.S. is focusing on increasing the resilience against growing threats and implementing policies to effectively respond to these challenges [126].

Space Policy Directive-5 (SPD-5) [127], recently established by President Donald J. Trump, is the first comprehensive cybersecurity policy for space systems in the United States. It outlines key cybersecurity principles to protect space systems, which are crucial for functions like global communications, navigation, scientific observations, exploration, weather monitoring, and national defense that are vulnerable to malicious activities that could disrupt or destroy them, posing risks to critical national infrastructure. SPD-5 aims to foster practices within the U.S. Government and commercial space operations to shield space assets and their infrastructure from cyber threats. It aligns with the national cyber security strategy, emphasizing the protection of space assets from evolving cyber threats and preventing the creation of harmful space debris due to malicious activities. The directive advocates for integrating cybersecurity throughout all phases of space system development and emphasizes prevention, active defense, risk management, and sharing best practices.

**China:** Since the 2000s, China has experienced significant economic growth, which has enabled it to invest astronomical sums in its space program and develop various military-purpose satellites. The primary goal of China’s current policy is to realize President Xi Jinping’s “Chinese Dream”, focusing on developing and launching all space-related technologies independently. To achieve this, China has developed its doctrine and strategy [50].

China publicly advocates for the peaceful use of space and actively seeks international agreements for non-weaponization through the United Nations. Despite this stance, it continues to enhance its counter-space weapons capabilities, including integrating cyberspace, space, and electronic warfare (EW) into its military operations. This strategy evolution aligns with the People’s Liberation Army (PLA)’s view of space superiority as a crucial element in conducting “informatized warfare”, a concept that emphasizes control over information in space to deny adversaries intelligence and communication advantages [128]. China’s focus on space operations and counter-space capabilities, which has significantly accelerated since the 1980s, is partly influenced by observing the U.S. military’s space-based operations. The PLA perceives space as a key enabler for its forces and likely views counter-space measures as a strategic tool to deter or counter U.S. involvement in regional conflicts. This approach includes targeting reconnaissance, communication, navigation, and early warning satellites to disrupt adversaries’ capabilities.

**Russia:** Unlike China, Russia has been engaged in space competition with the United States since the Soviet era and has developed a variety of technologies over the years. Therefore, for Russia, maintaining superiority in space and space communication systems was an important factor in the past and continues to be so today [129].

Russia publicly advocates for space arms control agreements to prevent the weaponization of space but simultaneously views space as a critical warfighting domain and believes achieving supremacy in space is key to winning future conflicts. The Russian military doctrine recognizes the expanding importance of space due to its role in precision weapons and satellite-supported information networks. Despite expressing concerns about space weapons and seeking legal agreements to limit U.S. dominance in space, Russia is actively developing a wide array of counter-space capabilities to target U.S. and allied assets. As part of its military modernization, Russia is integrating space services into its armed forces and has over 60 years of technical experience in space. However, Moscow is wary of over-reliance on space for its national defense and has developed terrestrial redundancies for space services potentially denied during wartime. Russia perceives the U.S.’s missile defense and precision-strike capabilities, enabled by space technologies, as a threat to strategic stability and views the U.S.’s dependence on space as a vulnerability to exploit. The Russian counter-space doctrine includes the use of ground-, air-, cyber-, and space-based systems to target adversary satellites with a range of actions, from jamming and sensor blinding to destruction, aiming to deter aggression and control escalation in conflicts by selectively targeting adversary space systems [128].

**Summary:** Recently, the international situation has been unstable. For example, a second Cold War, primarily between the United States and China, is emerging more than it has done in the past, and the ongoing war between Russia and Ukraine continues to fuel this sense of instability. Consequently, it appears that worldwide strategies and policies are focused on protecting each country’s satellite communication systems while imposing checks on the satellite communication systems of adversary nations. Especially now that satellite communication systems are playing an important role in various fields, the strategies and policies of each country will become more important elements that directly influence the security of satellite communication systems.

## 7. Future Research Directions

Following our examination of attacks and defenses, this section explores potential security issues in future SCSs within fields such as 6G, various environments and standard protocols, AI-integrated SCSs, and quantum communications.

### 7.1. Standardized Terrestrial–Space Network Integration Protocols

SCSs utilize a variety of protocols, ranging from those traditionally used in terrestrial networks to those specifically designed for SCSs. Among the protocols currently recognized as standards are CCSDS [130], 3GPP NTN [131] and DVB-S [132].

The Consultative Committee for Space Data Systems (CCSDS) is a key organization in the development of communication and data system standards for spaceflight. The CCSDS has developed over 100 standards that have been adopted by space missions globally to ensure reliable and efficient space communication [130]. In parallel, the 3GPP NTN (non-terrestrial network) protocol framework, designed by the Third Generation Partnership Project (3GPP), aims to extend mobile network communication to satellite networks. The efforts of the 3GPP in Releases 17 and 18 regarding non-terrestrial networks (NTNs) mark a major milestone in the integration of SCSs with the 5G ecosystem. Specifically, Release 17 emphasizes the 3GPP’s focus on enabling satellite access for new radio (NR) in the FR1 bands, which are targeted at handheld devices, and on supporting massive IoT use cases with NB-IoT and eMTC technology [131]. Complementing these efforts, GSE (generic stream encapsulation) is a protocol for encapsulating streams defined by the digital video broadcasting (DVB) group, designed to carry packet-oriented protocols like IP over unidirectional PHYs such as DVB-S2, DVB-T2, and DVB-C2. The support provided by GSE includes multi-protocol encapsulation, network layer function transparency, multiple addressing modes, payload frame fragmentation over base band frames, and hardware filtering [132].

Despite the establishment of these standards, it is rare for only one protocol to be exclusively used. Some systems may employ proprietary protocols that differ from the established standards. Therefore, the development and application of integrated protocols are crucial for enabling efficient communication among diverse SCSs. This approach could also enhance interoperability between various SCSs.

### 7.2. Software-Defined Satellite Networks

Managing SCSs can be challenging due to their highly dynamic nature, such as various topologies and intermittent links. One of the significant difficulties faced is the cost and effort required for reconfiguration and resource allocation [133,134]. To address this issue, some researchers have suggested using software-defined network (SDN) features, which include flexible scalability, virtualization, and interoperability [135].

More specifically, research on software-defined satellite networks (SDSNs), which are SDN-enabled SCSs, has been proposed, such as Dijkstra and dynamic algorithms for enhancing network QoS and security reliability in SCSs [136,137,138]. Also, a few studies [139,140,141,142] have proposed a new load balancing algorithm to reduce congestion and bottlenecks in an SCS network. These studies can be applied to SCS security to mitigate DDoS attacks in an SCS network.

These approaches are particularly beneficial in managing the complex, dynamic, and heterogeneous nature of modern satellite constellations. However, there is still room for improvement. First, integrating SDSNs with terrestrial SDN systems presents significant challenges due to the differing technological demands and the unique requirements of space-based infrastructure. Additionally, as satellite constellations, such as Starlink, have been expanded to include thousands of satellites, the complexity and demand for efficient management of these vast networks have increased considerably. Lastly, the inherent issues of a variable latency and link instability in satellite communications can further impede the performance of SDSNs, potentially degrading the quality of service and the overall network reliability [133].

In particular, the integration of ground and satellite networks through SDSNs is crucial for creating a unified global network that can seamlessly support various applications, from global internet coverage to specialized services like disaster management communications. However, this integration is challenging due to the architectural and operational discrepancies between terrestrial and satellite networks. Moreover, SCSs inherently have a wide range of coverage due to their characteristics. This can make them vulnerable to attacks such as eavesdropping, requiring encrypted traffic during handover and upload/download processes. However, encryption in satellite communication systems faces difficulties due to high computational resource requirements and traffic loss.

A few studies [143,144] are attempting to address the above issues, but they still face challenges in resolving frequent handovers and delays, which are unique features of SCSs.

### 7.3. AI-Based Satellite Communication Security

As attacks on SCSs become more advanced, traditional security methods, such as rule- or behavior-based methods, are no longer adequate for countering them. Identifying the attack patterns has become a challenge, and in response, security methods based on AI have been developed, since AI-powered systems are capable of finding more sophisticated patterns that are difficult for humans to detect. Therefore, it is thought that AI will play a significant role in the next generation of SCS security methods [145,146].

In signal management, ML-powered methods that distinguish benign signals from malicious signals in SCSs have been proposed. For example, ref. [147] introduces a novel ML methodology designed to automatically detect both short-term and long-term interferences within satellite communication spectra in real time. Similarly, ref. [148] proposes a deep-learning-based system for managing interference, a significant issue in both terrestrial and satellite networks. These ML techniques enhance the accuracy of detecting carrier signals in the frequency domain, which is vital for securing communication channels against unauthorized access and interference. On the other hand, a few studies [149,150,151] have suggested mitigating anti-jamming using AI based on signal management. These studies focused on enhancing real-time interference detection using ML to block jamming of SCSs.

Additionally, optimizing ISL communications using deep reinforcement learning methods makes SCSs more robust and secure [145,152]. These methods help in optimizing routing and satellite selection, thereby enhancing the overall security posture by ensuring stable and secure satellite connections and mitigating eavesdropping attacks.

Lastly, AI techniques can be leveraged to forecast and manage network traffic in satellite communications, which includes preventing potential cyber attacks and ensuring the integrity of data transmission. Neural networks and other AI models aid in proactive security management, adapting dynamically to new threats. For example, AI-based network intrusion detection technologies used in terrestrial networks could be applied. If the existing models are adapted and made more lightweight for satellite communication systems, it could be possible to defend against various attacks [153]. Recently, federated learning (FL) has emerged as a promising direction for security. FL, as a new method that protects user privacy and data privacy, could potentially replace traditional ML and DL methods. Utilizing FL could enhance the overall network availability of satellite communication networks through traffic classification [154,155].

Leveraging AI technologies has enhanced SCS’s security in various fields, such as signal management, ISL communication optimization, and traffic management. However, AI can be utilized for more sophisticated attacks on SCSs. For instance, in satellite communication systems, attacks could involve the application of encrypted traffic classification methods, e.g., ET-BERT [156], to execute fingerprinting attacks on satellite communication traffic. Satellite communication systems broadcast signals via RF, which makes it easy to randomly collect traffic data and build datasets, which could facilitate various attacks on these systems [157,158,159].

In addition to the issues mentioned above, AI-powered SCSs also have limitations. For example, AI in SCSs brings limitations such as a limited access to and a reduced quality of data, which can be noisy and incomplete, making it challenging to develop and train accurate models. Additionally, the computational resources available on satellites are often constrained [160], which can limit the efficiency and feasibility of advanced AI algorithms. The reliability and robustness of AI systems must also be maintained in extreme operational environments; however, AI models may falter when faced with new situations or anomalies not present in the training data [14].

### 7.4. Full Quantum Networks

SCSs, a crucial national asset, have become a target for eavesdropping attempts by enemies. However, traditional cryptography methods are ineffective due to technical and resource limitations [161]. One proposed possible solution is applying the quantum internet principle, where packet information becomes unreadable to eavesdroppers.

According to recent research, the development of quantum networks can be outlined in five stages. The prepare and measure networks stage enables end-to-end QKD without the need for intermediary nodes, which is essential for basic quantum communication tasks. The entanglement distribution network stage involves the deterministic or heralded creation of entangled states between nodes without requiring quantum memory, crucial for enhancing the reliability and functionality of the network. Quantum memory networks introduce local quantum memory at the nodes, enabling the storage and manipulation of quantum states and supporting sophisticated operations and protocols for complex distributed quantum applications. The few-qubit fault-tolerant network stage allows for fault-tolerant quantum operations with a few qubits, significant for tasks requiring high reliability and for applications requiring prolonged quantum state coherence and manipulation. Finally, the quantum computing network stage represents a fully developed quantum internet, where quantum computers are interconnected, facilitating the unrestricted exchange of quantum information and the execution of computationally intensive quantum algorithms. This stage realizes the ultimate goal of quantum networking, enabling a vast array of quantum applications that are unfeasible with classical technologies [162,163].

For the quantum internet, the essential components include physical quantum channels, quantum repeaters, and end nodes/quantum processors. Physical quantum channels are important for transmitting qubits over distances, where minimizing the photon loss and managing the inherently lossy nature of quantum communication are critical [164]. The quantum repeaters are needed to combat photon loss and the exponential scaling of loss with distance. They are strategically placed along the channel to effectively extend the reach of quantum communication and may also serve as long-distance routers within quantum networks. Also, end nodes/quantum processors range from simple devices capable of preparing and measuring single qubits to sophisticated large-scale quantum computers. These nodes are vital for various quantum network tasks and must have robust storage for quantum states and high-fidelity quantum-information-processing capabilities, along with compatibility with photonic communication hardware, especially for efficient interfacing with light at telecom bands [163,165,166].

However, currently, due to technical limitations, quantum communication in SCSs transmits only quantum cryptographic keys through quantum links, while data are sent through classical channels. Recently, research on satellite-based quantum repeaters has been initiated, but it cannot keep up with the speed and stability of classical communication networks [167]. Consequently, the data still utilize the existing communication infrastructure, sharing the same vulnerabilities inherent in current telecommunication equipment and software. Therefore, the final goal of quantum communication is to have a network entirely composed of quantum communication systems. This means a network made solely of quantum processors and quantum repeaters, but as of now, a network solely based on this communication system has not yet been developed. Recent research has shown success in a prototype city-scale quantum network where eight users, each connected in a mesh network, successfully communicated in the experiment [168]. Despite quantum networks being in the experimental phase and facing many limitations, it is expected that they will eventually evolve from QKD in satellite networks to a fully quantum mesh network. Furthermore, through these advancements, we can expect a significant increase in security for satellite communication systems.

### 7.5. Discussion

Given the complex and evolving landscape of SCSs, prioritizing areas for research and development is crucial for stakeholders aiming to enhance security and functionality. Considering the maturity and development speed of technology, there are several research areas that should be prioritized at present.

AI is considered to be the most important field in satellite communications. This is because AI is currently the fastest developing field, and at the same time, it makes the greatest contribution to SCS security. Therefore, leveraging AI in satellite communication could revolutionize security measures against sophisticated threats such as jamming and eavesdropping. However, this requires careful consideration of AI’s vulnerabilities and the unique constraints of satellite environments.

The development of SDSNs is another area of priority. By centralizing control and enhancing network resource allocation, SDSNs could dramatically improve the management of satellite constellations, addressing challenges like the link instability and variable latency. This is particularly important as the scale of networks like Starlink grows. However, the biggest problem with SDSNs is that integration with terrestrial networks is not easy, and this is thought to be a problem in a similar context to the integration of ground-space protocols.

Lastly, advancing quantum communication technologies in SCSs holds promise for unprecedented security capabilities, although it is still in its early stages and requires substantial foundational research. Addressing these areas effectively would require a coordinated approach, balancing technological innovation with rigorous security practices to safeguard this critical infrastructure against both current and emerging threats.

## 8. Conclusions

In this paper, we have comprehensively classified potential attacks on satellite communication systems (SCSs), examining the unique characteristics and vulnerabilities associated with each type. In particular, the broad coverage and wireless nature of SCSs not only enhance service accessibility but also increase the susceptibility to severe threats such as DoS and DDoS attacks. These attacks pose the highest risk due to their potential to completely disrupt services. Additionally, the challenges associated with the maintenance of these systems heighten their vulnerability to eavesdropping and spoofing, compromising the integrity and confidentiality of communications.

Through a sophisticated classification framework, we evaluate the existing defenses against these threats, such as blockchain, cryptography, and quantum communication methods, and identify critical areas. Additionally, we explore and summarize the policies of various countries. Our investigation into the policies of various countries has revealed a diverse range of approaches, reflecting differing national priorities and capabilities, which highlights the need for international cooperation.

Finally, we compiled a list of potential future security issues and provided directions for future research, such as AI, integrated protocols, SDNs, and quantum communication.

## Figures and Tables

**Table 1:** Comparison of related surveys.

Ref.	Security Attack				Security Defense					Policy and Strategy	Future Directions
	**Eaves-** **Dropping**	**Jamming and** **Spoofing**	**DoS and** **DDoS**	**Energy**	**PLS**	**Blockchain**	**SDN**	**Crypto and** **QKD**	**Anti-** **Satellite**		
[8]	✓				✓						✓
[9]	✓				✓						✓
[10]	✓	✓	✓		✓						✓
[11]		✓	✓			✓					✓
[12]	✓				✓		✓				
[13]								✓			✓
[14]	✓	✓	✓		✓			✓			✓
[15]		✓	✓					✓			✓
[16]	✓	✓	✓		✓	✓		✓			✓
[17]		✓			✓			✓			✓
[18]	✓	✓	✓		✓			✓			✓
This Survey	✓	✓	✓	✓	✓	✓	✓	✓	✓	✓	✓

**Table 2:** Comparison of satellite constellation.

Feature	LEO	MEO	GEO
Altitude	500~1200 km	2000~20,000 km	36,000 km
Propagation Delay	Approx. 30 ms	Approx. 125 ms	Approx. 600 ms
Earth Coverage	Small	Large	Very Large
Data Loss	Low	Low	Medium
Required Satellite	At Least Tens to Hundreds	At Least 6	At Least 3
Gateway	Numerous Flexible	Local Flexible	Stationary
Orbital Period	Approx. 85~120 min (11~17 round per day)	Approx. 120~480 min	24 h

**Table 3:** Comparison of eavesdropping attack methods.

Characteristic	Passive Eavesdropping	Active Eavesdropping
Victim Awareness	Impossible	Possible
Impact in	Personal Information	Cryptography (Decryption)
Technical difficulty	Easy	Need Sophisticated Hardware or Software
Mitigation	Easy	Hard

**Table 4:** Pros and cons of various defense techniques for jamming attacks in SCSs.

Solution Type		Pros	Cons
*Anti-* *Jamming* *Techniques*	Game theoretic approaches	Uses advanced technologies like spread spectrum, frequency hopping, and robust coding. Provides strategic defense mechanisms.	Can be complex and costly to implement. Game-theoretic models may need extensive computation.
	Filtering techniques	Enhances signal resilience against interference. Effectively isolate jamming signals.	Requires sophisticated signal processing capabilities. Filtering may not be fully effective in highly congested environments.

**Table 5:** Pros and cons of various mitigation techniques for DoS/DDoS attacks in SCSs.

Solution Type		Pros	Cons
*DoS/DDoS* *Mitigation*	Blockchain- Based Mitigation	Provides transparency and security via immutable record keeping. Enhances node stability and detection of alterations. Effective against cyber-physical and data integrity attacks.	Blockchain complexity might introduce latency. High energy consumption and computational requirements. Dependency on the robustness of the underlying blockchain technology.
	Secure- Routing- Based Mitigation	Protects against internal and external malicious attacks on routing. Trust-based algorithms improve security and network performance. Adaptable to dynamic conditions and varying levels of threat.	May require frequent updates and maintenance. Depends on accurate trust and risk assessment models. Potential scalability issues in large satellite networks.
	SDN- Based Mitigation	Offers network flexibility and efficient resource management. Rapid detection and response to DDoS through optimized algorithms. Facilitates energy-efficient network operations.	Relies on the reliability of SDN controllers which can be a single point of failure. Requires substantial initial setup and configuration. Possible security vulnerabilities in the SDN architecture itself.
	Collaborative- Based Mitigation	Leverages multiple nodes for enhanced detection and mitigation. Uses blockchain and AI for robust, adaptive defenses. Suitable for complex, multi-domain environments.	Coordination among diverse systems can be challenging. High overhead for maintaining collaboration mechanisms. Involve privacy and data sharing concerns among stakeholders.

## Data Availability

Data sharing is not applicable.

## Abbreviations

The following abbreviations are used in this manuscript:

AI	Artificial Intelligence
BMD	Ballistic Missile Defense
cFS	Core Flight System
CSTN	Cognitive Satellite–Terrestrial Integrated Networks
CTI	Cyber Threat Intelligence
DCED	Distributed Collaborative Entrance Defense
DDoS	Distributed Denial of Service
DL	Deep Learning
DOA	Direction of Arrival
DoS	Denial of Service
DPD	Defense Plane Digest
DSSS	Direct Sequence Spread-Spectrum
DTN	Delay-Tolerance Network
DVB	Digital Video Broadcasting
DVB-S	Digital Video Broadcasting—Satellite
ECN	Explicit Congestion Notification
ETD	Entrance Traffic Digest
FHSS	Frequency Hopping Spread Spectrum
FSS	Fixed Satellite Service
GEO	Geostationary Earth Orbit
GMR	GEO-Mobile Radio
GNSS	Global Navigation Satellite System
GPS	Global Positioning System
GSE	General Stream Encapsulation
HAPS	High-Altitude Platform Station
HELLADS	High-Energy Liquid Laser Area Defense System
HTTP	Hypertext Transfer Protocol
IoT	Internet of Things
LEO	Low Earth Orbit
LiDAR	Light Detection and Ranging
MEO	Medium Earth Orbit
MIRACL	Mid-Infrared Advanced Chemical Laser
MISO	Multiple-Input Single-Output
ML	Machine Learning
MSNET	Mobile Satellite Communication Network
NFV	Network Function Virtualization
NMA	Navigation Message Authentication
NR	New Radio
NTN	Non-Terrestrial Network
OCS	Onboard Computer System
OPSPF	Orbit Prediction Shortest Path First
PD	Power Distortion
PHY	Physical Layer
PLA	People’s Liberation Army
PLS	Physical Layer Security
QKD	Quantum Key Distribution
QoS	Quality of Service
SAR	Synthetic Aperture Radar
SATCOM	Satellite Communication
SCA	Spreading Code Authentication
SCS	Satellite Communication System
SDN	Software-Defined Network
SDSN	Software-Defined Satellite Network
SDR	Software-Defined Radio
SLT	Secure LEO Trust
SM-3	Standard-Missile 3
SQM	Signal Quality Monitoring
SSL	Secure Socket Layer
STIN	Satellite–Terrestrial Integrated Network
SVM	Support Vector Machine
TDOA	Time Difference of Arrival
TESLA	Timed Efficient Stream Loss-Tolerant Authentication
TLS	Transport Layer Security
TOS	Type of Service
VoIP	Voice over Internet Protocol
